# MSstatsTMT Improves Accuracy of Thermal Proteome Profiling

**DOI:** 10.1016/j.mcpro.2025.100999

**Published:** 2025-05-27

**Authors:** Amanda M. Figueroa-Navedo, Rohan Kapre, Tushita Gupta, Yingrong Xu, Clifford G. Phaneuf, Pierre M. Jean Beltran, Liang Xue, Alexander R. Ivanov, Olga Vitek

**Affiliations:** 1 Barnett Institute of Chemical and Biological Analysis, Department of Chemistry and Chemical Biology, Northeastern University, Boston, Massachusetts, USA; 2 Khoury College of Computer Science, Northeastern University, Boston, Massachusetts, USA; 3 Discovery Sciences, Pfizer Inc, Groton, Connecticut, USA; 4 Sanofi, Disease Profiling and Functional Genomics, Cambridge, Massachusetts, USA; 5 Machine Learning and Computational Sciences, Pfizer Inc, Cambridge, Massachusetts, USA

**Keywords:** thermal proteome profiling, non-parametric response curves, MSstats, MSstatsTMT, proteomics

## Abstract

Thermal proteome profiling investigates protein–protein, protein–nucleic acid, or protein–drug interactions, and the impact of metabolite binding and post-translational modifications on these interactions. The experiments quantitatively characterize biological samples treated with small molecules *versus* controls and subjected to timed exposures to multiple temperatures. Typically, each enzymatically digested sample is labeled with a tandem mass tag (TMT), where each TMT channel corresponds to a specific temperature treatment, and profiled using liquid chromatography coupled with mass spectrometry in data-dependent data acquisition mode. The resulting mass spectra are processed with computational tools to identify and quantify proteins and filter out noise. Protein-drug interactions are detected by fitting curves to the protein-level reporter ion abundances across the temperatures. Interacting proteins are identified by shifts in the fitted curves between treated samples and controls. In this article, we focus on data processing and curve fitting in thermal proteome profiling. We review the statistical methods currently used for thermal proteome profiling and demonstrate that such methods can yield substantially different results. We advocate for the statistical analysis strategy implemented in the open-source R package MSstatsTMT, as it does not require subjective pre-filtering of the data or curve fitting and appropriately represents all the sources of variation. It supports experimental designs that trade-off temperatures for a larger number of biological replicates and handles multiple drug concentrations or pools of samples treated with multiple temperatures, thus increasing the sensitivity of the results. We demonstrate these advantages of MSstatsTMT as compared to the currently used alternatives in a series of simulated and experimental datasets, which include conventional thermal proteome profiling and its OnePot counterpart that pools the samples treated at multiple temperatures into one sample and incorporates multiple doses of a drug. The suggested MSstatsTMT-based workflow is documented in publicly available and fully reproducible R vignettes.

## Highlights

• Thermal proteome profiling pool designs were evaluated with four statistical models.
• The choice of processing and statistical modeling choice affected the results.
• MSstatsTMT steps for thermal profiling and pooled designs are detailed in R vignettes.
• Recommended workflows for MSstats processing and statistical modelling are described.

## Graphical Abstract

**Figure:**
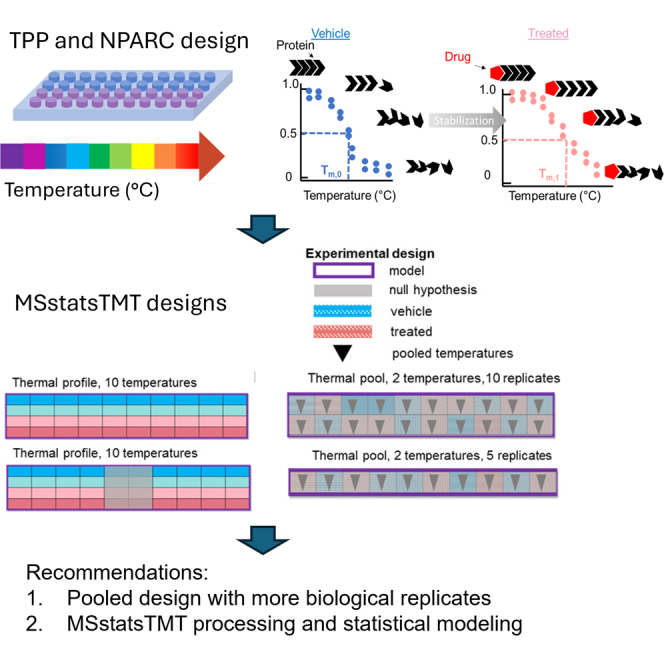

## In Brief

Figueroa-Navedo *et al*. evaluate differences in processing and statistical modeling and how the selection affects the results with several published datasets. Their data show that pooled designs with MSstatsTMT processing allow for additional biological replicates and greater reproducibility. Their findings are complemented with step-by-step R vignettes.

Cellular-level target and off-target engagement is an important step for drug target validation and drug development (1, 2, 3, 4, 5, 6). Since past challenges involving target engagement have negatively impacted the success of clinical trials (7, 8, 9), Western blot-based cellular thermal shift assays (CETSA) were introduced to characterize target engagement in cells or tissues (4, 10, 11). CETSA treats biological samples with small molecules *versus* controls and subjects them to repeated, timed exposures to multiple temperatures. The Western blots were later replaced with mass spectrometry-based thermal proteome profiling (TPP), where each treated sample is enzymatically digested and labeled with a tandem mass tag (TMT). The samples are combined into one or a small number of TMT plexes. Features in the resulting mass spectra are processed using computational tools to identify peptide ions and proteins, and to quantify the reporter ion abundances at both the peptide-spectrum-match and protein levels (5, 12). Protein-drug interactions are then detected by statistically characterizing protein-level profiles of TMT reporter ion abundances across the temperatures (4, 13), and quantifying the differences in protein unfolding and aggregation with *versus* without the drug.

The statistical characterization of protein-level profiles is an important and challenging step of the process. The systematic changes in protein profiles across the drug treatments and across the temperatures can be small. They can be obfuscated by confounding mechanisms such as protein-protein interactions, metabolite binding, nucleic acid-protein interactions, and protein-small molecule interactions (1, 2, 4, 5, 10, 12, 13, 14, 15, 16, 17, 18, 19, 20, 21, 22, 23, 24, 25, 26). They can be negatively impacted by artifacts such as the limited lifetime of the drug-compound complex or the suboptimal choice of temperatures. In addition to the systematic changes in the protein-level profiles, biological replicates, TMT plexes, and artifacts of spectral identification and quantification introduce random variation of reporter ion abundances (27, 28). Statistical methods must detect the systematic changes induced by the treatments and distinguish them from artifacts of random variation.

Early statistical methods for TPP experiments employed *ad hoc* criteria for filtering poor quality data, fit sigmoid curves to the remaining protein profiles, and used subjective characteristics of the fit to detect changes in the protein profiles with and without the drug. These subjective criteria are inadequate for characterizing the error rates associated with the decisions (21, 29). More recently, non-parametric analysis of response curves (NPARC) (5) proposed a more principled full-scope statistical approach that framed the goal of detecting changes as a statistical hypothesis testing. However, the method has its own limitations, including numeric instability and inability to distinguish biological (between-replicate) and technological (within-replicate) sources of variation.

To further improve upon the throughput and the sensitivity of thermal proteome profiling, more advanced experimental designs have recently been proposed. Some designs, such as 2D-TPP, expand the space of the treatments to support a range of drug concentrations in addition to a range of temperature treatments (1, 2, 12, 16, 17, 18). These designs increase the information content in the data, at the expense of the experimental and data analysis complexity (1). Alternatively, OnePot designs compress the space of the treatments by pooling aliquots of treated samples heated at multiple temperatures before TMT labeling and mass spec analysis (18, 19, 20). The design is flexible in terms of the number of temperatures pooled, the number of biological replicates, the number of TMT plexes, and the sample processing time (18, 20, 23, 24), but can be negatively impacted by an inadequate choice of temperature ranges. These designs can be combined, *e.g.*, by combining a range of drug concentrations with OnePot pooling of samples treated with different temperatures (18). Unfortunately, there is currently a limited understanding of appropriate statistical analysis strategies for these complex designs.

This article focuses on the statistical analysis step of thermal proteome profiling experiments. We review the currently used statistical methods and demonstrate that different reasonably suitable methods can yield substantially different results. We advocate for the statistical analysis strategy implemented in the open-source R/Bioconductor package MSstatsTMT (27, 28) as it side-steps the need for subjective pre-filtering of the data and curve fitting, and is numerically stable. Furthermore, the ability of MSstatsTMT to appropriately represent the structure of variation results in better-calibrated error rates. Finally, MSstatsTMT supports experimental designs that trade off temperatures for a larger number of biological replicates and extends to other experimental designs, such as a combination of a range of drug concentrations with OnePot pooling.

We demonstrate these advantages of MSstatsTMT in a series of simulated and experimental datasets, which include conventional thermal proteome profiling and its OnePot counterpart that pools the samples treated at multiple temperatures into one sample.

## Experimental Datasets

The proposed MSstatsTMT-based workflow and analyses of each dataset are documented in publicly available vignettes https://github.com/Cleanvalidation/MSstatsThermalProfile. The Proteome Discoverer search parameters are included in Experimental datasets: Data processing section in Supplemental Data.

### Dataset 1—Phaneuf *et al.*

#### Experimental Design

Phaneuf *et al.* (25) investigated method development strategies to improve drug-target identification in mass spectrometry-based thermal stability assays. In the original study, intact Jurkat cells were treated with a highly specific mitogen-activated protein kinase inhibitor CH4987655 at a concentration of 10 μM (30). The study had two conditions, vehicle and treated, two biological replicates per condition, and 10 temperatures. The samples were labeled with TMTpro (11 channels), mixed into four plexes (one per biological replicate), with channel 126 as the reference channel corresponding to the 37 °C temperature. Channel 131C was designated as a carrier channel and was discarded from further data processing. Experimental settings did not include fractionation. Mass spectra were acquired with an Orbitrap Exploris 480 in data-dependent acquisition mode at the MS^2^-level, as outlined in the original publication.

#### Evaluation Criteria

It has been previously shown using X-ray crystallography that the drug CH4987655 binds to MAP2K1 (30) (UniProt ID Q02750). Furthermore, MAP2K1 and MAP2K2 (UniProt ID P36507) have 81% sequence similarity, and the crystal structures of MAP2K1 shows evidence of interaction with the inhibitor in sites Lys97, Val211, Ser212 and Val127. Therefore, we view both MAP2K1 and MAP2K2 as known verified interacting proteins of drug CH4987655.

### Dataset 2—Leijten *et al.*

#### Experimental Design

Leijten *et al.* investigated the effects of 50 μM Napabucasin treatment on Zebrafish (*Danio rerio*) embryos (11). The experimental design consisted of two conditions, vehicle and treated, two biological replicates per condition, and 10 temperatures. The samples were labeled with TMT (10 channels) and mixed into four plexes (one per biological replicate), with a reference channel corresponding to 34 °C. Ten fractions were collected from each plex, and analyzed with a Q-exactive HFX mass spectrometer in data-dependent acquisition mode at the MS (2)-level with settings outlined in the original publication. In addition to the biological investigation, the authors used this dataset to develop and evaluate the NPARC data processing and statistical modeling procedures.

#### Evaluation Criteria

Napabucasin is often viewed as a Stat3 inhibitor; however, its mechanism requires further study (11, 31). For example, in the thermal profiling study by Leijten *et al*. conducted with NPARC, Stat3 (UniProt ID: Q6NV46) and Stat5 (UniProt ID: Q68SP3) had no evidence of protein interaction (11). At the same time, the study found that aldehyde dehydrogenases, expected oxidoreductase substrates of Napabucasin (gene IDs: aldh1a2, aldh1a2.1, aldh1a2.2, aldh1a3, UniProt IDs: Q90Y03, A2BGR9, F1QZU7, and Q0H2G3) were stabilized upon interaction with Napabucasin. In addition, the study found that another expected oxidoreductase substrate of Napabucasin Pora (UniProt ID: F1Q7F3) was destabilized, and Nqo1 (UniProt ID: F1QLV5) did not interact. However, a recent UniProt search on these accessions shows that since January 2023, most of these are viewed as obsolete except gene ID ALDH1a3 (UniProt ID: Q0H2G3), gene ID Stat3 (UniProt ID: Q6NV46), and gene ID Stat5 (UniProt ID: Q68SP3). Due to this uncertainty, in this manuscript, we view target geneID Aldh1a3 (UniProt ID: Q0H2G3), gene ID Stat3 (UniProt ID: Q6NV46), and Stat5 (UniProt ID: Q68SP3) as the ground truth interactors.

### Dataset 3a Thermal - Xu *et al.*

#### Experimental Design

Xu *et al.* investigated the stability of proteins as a function of rates of oxidation (SPROX) (18) and TPP. K562 cells were treated with 20 μM of a well-studied pan-kinase inhibitor Staurosporine, and DMSO (18). Two biological replicates per condition were treated with temperatures 37.3, 40.2, 44.9, 47.6, 51, 54, 57, 61.8, 64, 67 °C, and 75 °C. The samples were labeled with TMT11plex and mixed into four plexes (one per biological replicate) with a reference channel set at 37.3 °C. Twenty-four fractions were collected from each plex and analyzed with an Orbitrap Fusion Lumos Tribrid Mass spectrometer in data-dependent acquisition mode at the MS^3^-level. Additional settings were as outlined in the original publication.

#### Evaluation Criteria

We compared the results of thermal proteome profiling to a reference set of 522 human protein kinases reported in KinHub (32).

### Dataset 3b OnePot - Xu *et al*

#### Experimental Design

Xu *et al.* also investigated a OnePot counterpart to the conventional thermal profiling experiment (18). While the thermal profiling experiment only considered two conditions, the DMSO control and 25× (20 μM) Staurosporine, OnePot included additional Staurosporine concentrations (1×, 5×, 10×, and 25× Staurosporine, *i.e.*, five conditions total), and the number of biological replicates was increased to three per condition. Unlike with the conventional thermal profiling, samples from the same biological replicate treated with a total of 10 temperatures within the range of 45 to 58 °C were pooled into one mixture prior to TMT labeling and allocated to a single TMTpro 16-plex. This single-plex study did not require a reference channel, and the 16th channel was left empty and discarded from further analyses. Mass spectra were acquired with 24 off-line high pH fractionation on the same Orbitrap Fusion Lumos Tribrid as in Dataset 3a in a data-dependent acquisition mode at the MS (3)-level. Additional settings were as outlined in the original publication.

#### Evaluation Criteria

We compared the results of the OnePot investigation to the same reference set as for Dataset 3a.

### Dataset 4: Computer Simulation—Thermal Profiling

#### Experimental Design

To evaluate the statistical methods in situations with known ground truth, we simulated thermal proteome profiling datasets with known systematic and random variation. Since MSstatsTMT accounts for multiple variance components, we used MSstatsTMT workflow (after protein-level summarization) as the starting point for the simulations. The pseudocode describing the simulation is in supplemental Fig. S4. First, to simulate varying extents of interactions viewed as ground truth, we considered three simulation templates, namely, a strong-interacting protein MAP2K2 from Dataset 1, a weak-interacting protein Sap18 from Dataset 2, and a non-interacting protein Stat3 from Dataset 2. The templates were the systematic parts of the MSstatsTMT model fit to the vehicle and treated groups for these proteins, performed after the MSstatsTMT processing and log_2_ transformation as described in supplemental Table S1. For the non-interacting protein, the template was constructed by averaging the fitted values of the vehicle and treated conditions.

Second, to simulate the non-systematic effects, we set the technological within-plex variation in the simulation to the median value of the residual error from the MSstatsTMT fit to all the proteins in Dataset 1, *i.e.* $\sigma_{error}^{2}=0.267$, and assumed the technological between-plex variation to be negligible. To investigate plausible ranges of biological between-replicate variation, we considered the distributions of intra-class correlation coefficient (ICC, *i.e.*, proportion of the total variation attributed to the biological variation) defined as

(1) $$\%\;of\;biological\;variability=\frac{\sigma_{bio}^{2}}{\left(\sigma_{bio}^{2}+\sigma_{error}^{2}\right)}$$

in all the experimental thermal profiling datasets in this manuscript, as illustrated in supplemental Fig. S5. Two representative values in the experimental datasets were ICC of 5% (approximately 25th quantile) and 40% (approximately 66th quantile), were selected. We therefore selected two values of $\sigma_{bio}^{2}$, 0.014 and 0.178, that correspond to these values of ICC. For each combination of ground truth template and % of biological variation, we simulated 1000 protein instances. The same simulations were then repeated by varying the number of biological replicates.

#### Evaluation Criteria

For strong and weak protein interactions, a higher number of proteins with *p* < 0.001 indicated a higher sensitivity. For the non-interacting protein, a higher number of simulation instances with *p* < 0.001 indicates a larger number of false positives. Better-calibrated statistical models were expected to have a uniform distribution of *p*-values in the case of the non-interacting template.

### Dataset 5: Computer Simulation—OnePot

#### Experimental Design

The same protein templates as in Dataset four were used to simulate datasets with alternative OnePot experimental designs. The simulations were performed as in Dataset 4, with a modification reflecting the fact that OnePot experiments physically mix proteins prior to labeling and sample processing. To mimic this pooling for each protein and condition, the simulated values of 10 temperatures (*i.e.* 37.3,40.2,44.9, 47.6, 51, 54, 57, 61.8, 64, and 67 °C) were unlogged, averaged over the values corresponding to temperature treatments being pooled, and log_2_-transformed the average values back prior to the statistical inference. The simulations varied the number of pooled temperatures and the number of biological replicates, to illustrate their trade-off. The full pseudocode describing the simulation is presented in supplemental Fig. S6.

#### Evaluation Criteria

The evaluation criteria for the simulated OnePot experiments were the same as for the simulated thermal profiling experiments.

## Background

### Data Structures and Notation

In this section, we review the data structures and data analysis methods relevant to thermal proteome profiling. Consider a representative LC-MS/MS experiment with a 10-plex TMT labeling, where a plex contains all the samples from a biological replicate treated with either vehicle or drug, and with different temperatures. For analyses that start with protein-level summaries of reporter ion abundances, determined, *e.g.*, by Proteome Discoverer (PD), the data structure for one protein is an array of reporter ion abundances $Y_{pcrt}^{PD}$, where *p = 1*,…,*P* are *proteins*, *c* = 0,1 are *conditions*, *r* = 1,…, *R* are *biological replicates* (or *subjects*), and *t* = 0, …,*T* are discrete *temperatures*. *t* = 0 denotes the reference channel (when present) or the lowest temperature that can be used for normalization. supplemental Table S1*A* illustrates this data structure for the special case of one protein, *c* = 0 for vehicle and *c* = 1 for the drug-treated samples*, r* = 1, 2, and *t* = 0,…,9.

For analyses that start from peptide-spectrum-matches (also called *feature* level in the literature) (27, 28), the data structure for one protein is an array of feature intensities $X_{pcrti}^{PD}$, where *p = 1*,…,*P* are *proteins*, *c* = 0, one are *conditions*, *r* = 1,…,*R* are *biological replicates* (or *subjects*), *t* = 0,…,*T* are temperatures, and *i* = 1,…,*I* are features. supplemental Table S1*B* illustrates this data structure for one protein in the same experiment as above, with *I* = 2.

The first step in data analysis is processing; these data structures to prepare them for statistical modeling as described below.

### Data Processing

#### Data Processing Methods for Thermal Proteome Profiling

##### TPP and NPARC Processing

TPP and NPARC are Bioconductor packages specifically designed and frequently used for analyses of thermal profiling experiments (21). Here, we consider TPP v3.26.1 and NPARC v1.10.1 and follow their implementations at https://github.com/DoroChilds/TPP and https://git.embl.de/childs/TPP-data-analysis. NPARC recommends the user to perform the same data processing strategy outlined in supplemental Table S1 as TPP. First, the protein abundances $Y_{pcrt}^{PD}$ are exported from each plex with a data processing tool such as Proteome Discoverer (supplemental Table S1, Step 1). Next, to place all the thermal profiles on a comparable scale, the abundance of each protein across all the temperatures is normalized relative to its abundance in the reference channel (supplemental Table S1, Step 2). We denote the ratioed abundances ${\;Y^{\prime }}_{pcrt}$. Steps 3 to 5 in supplemental Table S1 are not relevant to TPP or NPARC processing. Finally, an additional Step 6 normalizes all the proteins using a subset of proteins present in all replicate experiments to improve quantification accuracy at higher temperatures (5), as summarized in supplemental Fig. S7 and detailed below.

To achieve the normalization in Step 6 in supplemental Table S1, TPP and NPARC select a subset of proteins that best represent the thermodynamic unfolding of the native-state of the protein at lower temperatures, and its full aggregation at higher temperatures. This subset, called “NormSet”, is used for normalization purposes only and does not constitute a permanent filtering of proteins in the dataset. To construct NormSet, the methods filter out proteins with undesirable properties, such as thermostability or resistance to unfolding, which manifest themselves by deviations from the defined ratios of higher temperatures to the lowest temperature. Specifically, the methods only keep in the subset the proteins with a pre-defined range of ratios of seventh, ninth, and 10th temperatures (t∈{6,8,9}, respectively) to that of the reference channel (when available) or the lowest temperature (*t* = 0), present in all the replicates of both vehicle and treated conditions (33). These ratios at higher temperatures are selected to remove artifacts from thermostable or thermolabile proteins.

Next, the algorithm calculates the median protein abundances over the proteins in the normalization set, separately for each temperature, condition, and replicate. A sigmoid curve is fit to the medians separately for each condition and replicate, and as a function of time. The algorithm identifies condition *c∗* and replicate *r∗* that maximize the R^2^ of the sigmoid fit. The normalization factor $Q_{t}$ is calculated as a ratio of values on the fitted curve over median ratioed values for condition *c∗* and replicate *r∗*, separately for each time. Finally, Step 6 in supplemental Table S1 multiplies the ratioed values $Y_{pcrt}^{\prime }$ by $Q_{t}$ to produce normalized protein abundances. An example of the sigmoid fits considered for each replicate is shown in supplemental Fig. S8 (34).

Although NPARC & TPP are Bioconductor packages, both require manual data preparation by the user as indicated in supplemental Table S1. NPARC & TPP can take protein abundances as input from vendor software such as Proteome Discoverer, or open source tools such as MaxQuant, as long as the data are converted to the right format. Therefore, prior to Step 2, the data must be manually converted to Bioconductor's ExpressionSet class by the user. An alternative to data processing before TPP and NPARC protocols is to use Isobarquant, a Python package that filters out precursor ion signals close to the noise level (precursor-to-threshold or P2T < 4 and signal within the isolation window S2I < 0.5), infers protein abundances while mitigating peptide ion co-fragmentation, and outputs the filtered results in an ExpressionSet format. While NPARC vignettes and manuscript describe protein filtering with at least one unique peptide in vehicle and treated groups, this step is not automated by the package (21). Therefore, supplemental Table S1, step 3 shows no filtering implemented for NPARC.

### Other Data Processing Methods Relevant for Thermal Proteome Profiling

#### MSstatsTMT Processing

Although MSstatsTMT is not specifically designed for thermal proteome profiling, it can be used for processing and statistical analyses of LC-MS/MS experiments with TMT labeling (28) as outlined in supplemental Table S1. MSstatsTMT takes as input not protein abundances but an array of PSM-level reporter ion intensities $X_{pcrti}^{PD}$ (also called feature intensities) and proceeds with its own protein-level summarization. The feature intensities are log_2_ transformed in Step 2 to facilitate the downstream statistical modeling. An optional filtering step, which we applied in this article (Step 3), removes proteins with one feature, peptide feature plexes that have over three missing reporter ion intensities, and peptide features shared across protein groups.

The default global spectrum-level normalization of MSstatsTMT (Step 4) equalizes median reporter ion abundance across all the channels. However, since the abundance of all the proteins is expected to decrease across the channels as temperatures increase, this normalization is not appropriate for thermal profiling and should be set as FALSE. Step 5 summarizes all the peptide features of a protein observed in a same plex into a single value per protein per channel, by imputing missing channels with the Accelerated Failure Time (AFT) (35) model and fitting an additive Tukey median polish to the observed and the imputed values, as described in the MSstats manuscript (28). In an experimental design with multiple plexes, a normalization is needed because different plexes of the same sample may contain different peptide features, and protein-level summaries are not directly comparable between the plexes. Therefore, Step 6 implements protein-level normalization equalizing the log-transformed reference channel (or the lowest temperature, *t* = 0, when the reference channel is not available) to the median value of the reference channels for all plexes and all peptide features of the protein, and then applies the corresponding changes to the other channels.

Unlike TPP and NPARC, which require users to aggregate peptide features into protein-level summaries and format the data into ExpressionSets, MSstatsTMT has built-in converters that take as input peptide features from various vendors and open-sourced software (*e.g.* ProteomeDiscoverer, MaxQuant, SpectroMine, OpenMS, or FragPipe), automate Steps 2 to 4, and output *Y*_*pcrt*_ in a format that is compatible with MSstatsTMT.

### Statistical Modeling

Once the data structures are prepared, the next step is statistical modeling. The individual methods differ in the specifications of statistical models, model-based null hypotheses, post-processing filtering, and model-based inference, as summarized in supplemental Data, Table S2 and supplemental Table S2. Since all the models are fit to one protein at a time, we omit the subscript *p* from the notation.

### Statistical Models for Thermal Proteome Profiling

Here we describe statistical models implemented in TPP and NPARC for thermal proteome profiling.

#### TPP Model

The TPP methods assumed that the biomolecular system reaches equilibrium during thermal denaturation. According to denaturation theory (36), during the unfolding the protein loses its native structure, leading to aggregation or the formation of nonfunctional structure. Therefore, we expect a monotonic decrease in protein abundance (5, 16), and early versions of TPP (versions 3.0.0 or lower) fit the observed protein abundances with a sigmoid curve. However, some proteins, for example, thermostable proteins, may require a broader temperature range for denaturation than what is implemented in the experiment, and exhibit more diverse profiles that cannot be described by a sigmoidal curve (21, 37, 38). Moreover, some proteins can exhibit positive slopes, *e.g.* as an artifact exacerbated by weak lysis. In an example where a protein would stay attached to the DNA, both could be brought down into the insoluble fraction. As heat increases, there is the possibility to aid the release of the protein from the DNA, resulting in a temporary increase in the measured relative abundance. To overcome these issues, recent versions of TPP (3.0.0–3.28.0) in Github (33) include the option to use natural splines. We describe the spline-based approach following Kurzawa *et al.* (17) here, and the earlier sigmoid curve-based approach (5) in Supplemental Section 2.3 for completeness.

#### TPP Spline Model Assumptions

**Fig. 1:**
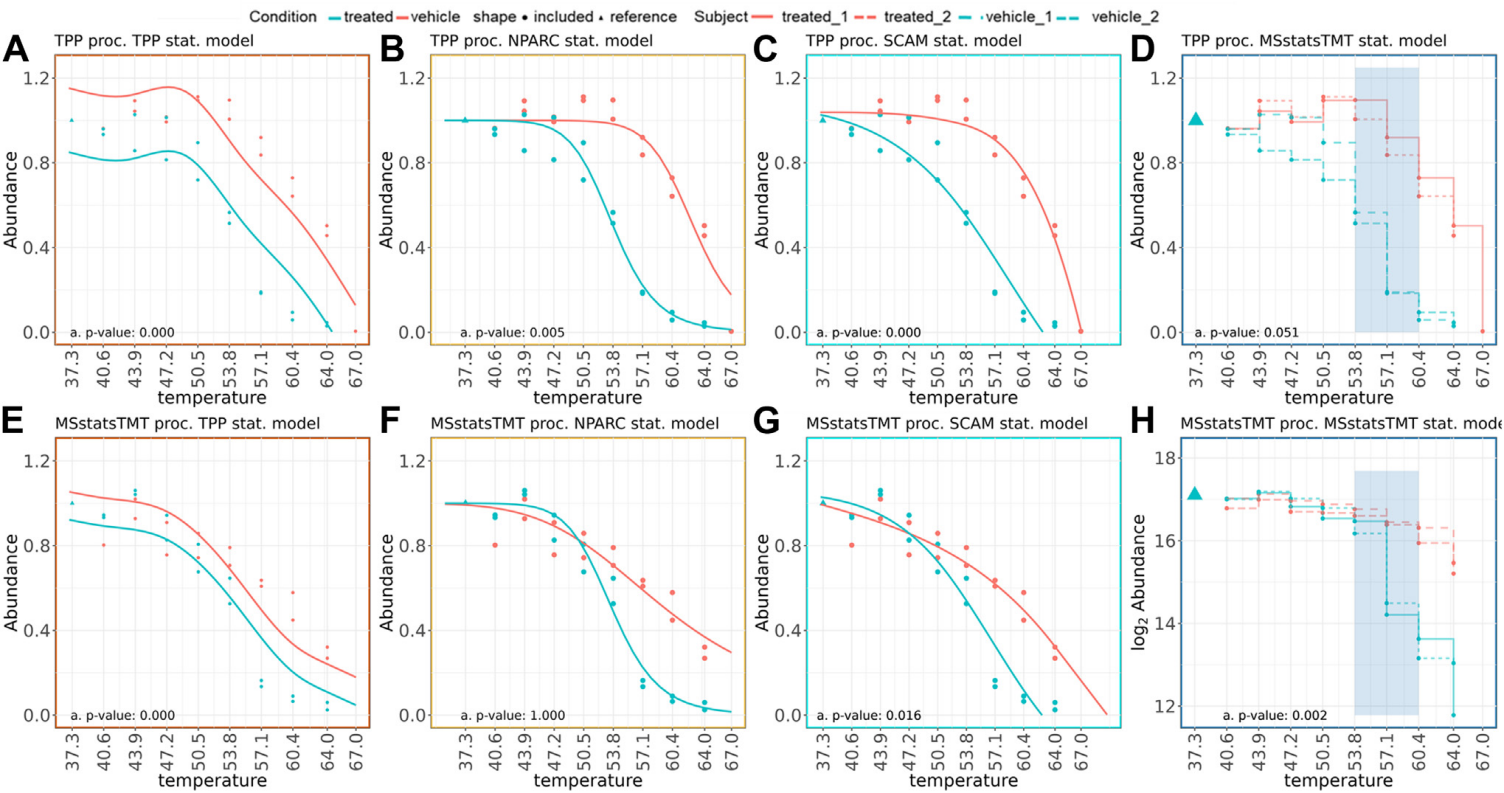
**Dataset 1- Phaneuf *et al.*, observed data and fitted curves for known interactor MAP2K1.***A–D*, TPP processing. We have outlined the recommended Thermal and OnePot designs in the vignettes under the recommended workflow folder in the repository. *E–H*, MSstatsTMT processing (supplemental Table S1, column 2; and vignette 2, section I). *A* and *E*, TPP statistical model with spline fit (Supplemental Data, Table 2, column 1); (*B* and *F*) NPARC statistical model (supplemental Data, Table 2, column 2); (*C* and *G*) SCAM statistical model (supplemental Table S2, column 3); (*D* and *H*) MSstatsTMT statistical model (supplemental Table S2, column 4). Line types emphasize the distinct biological replicates per each condition. Triangles indicate reference channels used for normalization by MSstatsTMT. *Blue areas* indicate subsets of temperatures used by the null hypothesis (supplemental Table S2, column 4, step 2). Different processing and modeling strategies produce different curves and different statistical testing results.

Step 1 of supplemental Table S2, Column 1 summarizes the spline model in TPP. The model represents the response $Y_{crt}$ as a linear combination $\sum_{b}s_{bc}\left(t\right)$ of non-linear basis functions *s*_*bc*_(*t*), characterized by degrees of freedom *d*. The default implementation iterates over several values of *d* to find the one optimizing Akaike’s Information criterion. Since this increases the processing time, the TPP repository recommends setting *d* = 4. The model is fit separately for each condition *c*. Figure 1*A* illustrates the TPP spline fit in the special case of protein MAP2K1 from Dataset 1, and supplemental Fig. S10*A* illustrates the same for protein MAP2K2 from Dataset 1. As can be seen, the spline curves are not restricted to be monotonic, and some temperatures can have a positive slope that may be biologically meaningful but also may be artifacts. Moreover, the model does not distinguish the variation between biological replicates from a residual within-replicate deviation from model fit.

#### TPP Null Hypothesis for the Spline Model

The null hypothesis is a reduced model, where the same curve simultaneously represents all the conditions (supplemental Table S2, Step 2), and all the other assumptions are unchanged.

#### TPP Model-Based Inference for the Spline Model

The test statistic in Equation 2 quantifies the discrepancy between the residual sum of squares of the fit of the reduced model (*RSS*_0_) and that of the full model (*RSS*_1_) in an *F* statistic, which under the null hypothesis follows the *F* distribution with *d*_1_ and *d*_2_ degrees of freedom. The degree of freedom *d*_1_ is the difference between the number of parameters in the full and in the reduced model. The degree of freedom *d*_2_ is the number of observations minus the number of parameters in the full model.

(2) $$F=\frac{\frac{RSS_{0}-RSS_{1}}{d_{1}}}{\frac{RSS_{1}}{d_{2}}}\overset{H_{0}}{∼}F\left(d_{1},d_{2}\right)$$

TPP enhances the test statistic above using Empirical Bayes moderation, popularized by *Limma* (39), which improves the precision of the variance estimation at the expense of introducing bias. It replaces the denominator in Equation 2 with a moderated version shown in (supplemental Table S2, Step 5, column 1). In the moderated version, ${\tilde{s}}_{i}^{2}$ is defined as

(3) $${\tilde{s}}_{i}^{2}=\frac{d_{0}s_{0}^{2}+d_{2}s^{2}}{d_{0}+d_{2}}$$

where *s*^2^ is the estimate of residual variance in the full model, and *d*_0_ and *s*_0_ are estimated empirically from all the proteins combined. The test statistic is compared to the F distribution with (*d*_1_, *d*_0_+*d*_2_) degrees of freedom. The *p*-values are adjusted for multiple testing with the method by Benjamini and Hochberg (40).

#### NPARC Model

NPARC model is a more recent approach proposed by the same group, that implements more formal inference for a sigmoid curve fit (21, 33).

#### NPARC Model Assumptions

The NPARC fits a sigmoid curve shown in supplemental Table S2, Step 1, column 2. The parameters of the TPP sigmoid equation are the lower plateau *p*, and constants *a* and *b*. Similarly to the more recent versions of TPP, the curves are fit separately for each condition but jointly for all the replicates. NPARC does not implement a monotonicity constraint, and does not distinguish the biological between-replicate variation from the lack of fit variation. Figure 1*B* illustrates the NPARC fit in the special case of protein MAP2K1 from Dataset 1, and supplemental Fig. S9*B* illustrates the same for protein MAP2K1 from Dataset 1.

#### NPARC Null Hypothesis

Similarly to TPP, NPARC specifies the null hypothesis in terms of a reduced model, where the same sigmoid curve is simultaneously fit to all the conditions (supplemental Table S2, Step 2, column 2).

#### NPARC Model-based Inference

At this stage, NPARC recommends keeping the inference proteins with high *R*^2^ and a lower sigmoid plateau (supplemental Table S2, Step 4, column 2). The inference starts with the same test statistic as for TPP (Equation 2); however, the approach differs in details of Empirical Bayes moderation that improves the estimation of variation. Specifically, the method separately approximates the numerator and the denominator of Equation 4 as:

(4) $$\frac{{{\;\text{RSS}}_{0}-RSS}_{1}}{d_{1}}\overset{H_{0}}{∼}\frac{\sigma_{0}^{2}}{d_{1}}\cdot \chi^{2}\left(d_{1}\right)\;\text{and}\;\frac{{RSS}_{1}}{d_{2}}\overset{H_{0}}{∼}\frac{\sigma_{0}^{2}}{d_{2}}\cdot \chi^{2}\left(d_{2}\right)$$

where the effective degrees of freedom *d*_1_ and *d*_2_, and the scale parameter $\sigma_{0}^{2}$ are the same for all the proteins and are estimated empirically from the entire dataset. The test statistic is compared to the *F* distribution with (*d*_1_, *d*_2_) degrees of freedom, and the *p*-values are adjusted for multiple testing with the method by Benjamini and Hochberg (40).

### Other Statistical Models Relevant for Thermal Proteome Profiling

Here we describe two additional statistical approaches, which, although not originally developed for thermal proteome profiling experiments, can be applied as summarized in supplemental Table S2. Both methods explicitly address the repeated nature of measurements of protein abundances for the same biological replicate across temperatures and the nonlinear nature of changes in protein abundance.

#### Shape-Constrained Additive Model (SCAM) for Experiments with Repeated Measures

Shape-Constrained Additive Models (SCAM) is a general statistical methodology, proposing an alternative specification of generalized additive models with splines (41, 42). Unlike the splines implemented in TPP, SCAM splines explicitly incorporate a monotonicity constraint to avoid overfitting in experiments where no increase in protein abundance is expected. They also distinguish the biological between-replicate variation and the technological within-replicate variation by introducing two distinct variance components. SCAM models are implemented in an R package by the same name, available on CRAN (43). Figure 1*C* illustrates the SCAM fit in the special case of protein MAP2K1 from Dataset 1, and supplemental Fig. S9*C* illustrates the same for protein MAP2K1 from Dataset 1. As can be seen, the SCAM splines do not have positive slopes and differ substantially from those of TPP.

#### SCAM Null Hypothesis

Unlike NPARC and TPP, the null hypothesis with SCAM is formulated in terms of differences in means (DIM) of thermal profiles between two conditions, on average over the temperatures (Step 2).

#### SCAM Model-Based Inference

The test statistic in Step 5 is derived using a standard large-scale approximation procedure for generalized additive models (41, 42), where the denominator accounts for the two variance components. Under the null hypothesis, the test statistic asymptotically follows a Standard Normal distribution. The *p*-values are adjusted for multiple testing with the method by Benjamini and Hochberg (40).

#### MSstatsTMT Model for Experiments With Repeated Measures

MSstatsTMT is specifically designed for proteomic experiments with repeated measurements, TMT labeling, and multiple plexes. It is implemented in an open-source R/Bioconductor package by the same name (27, 28). First, MSstatsTMT discards the reference channel *t* = 0, and models the remaining temperatures *t* = 1,…,*T*. Next, MSstatsTMT employs a family of linear mixed effects models, shown in supplemental Table S2, Step 1, column four for the case of the experimental design in this manuscript. Similarly to SCAM, MSstatsTMT distinguishes the biological between-replicate variation and the technological within-replicate variation by introducing two distinct variance components. Unlike all the methods above, MSstatsTMT does not attempt to fit a smooth curve to protein abundances across temperatures. Instead, it models the abundances semi-parametrically, such that each temperature has its own expected value, with no constraints. Therefore, the fit is not constrained by monotonicity or by a sigmoid shape, but is easy to fit and is numerically stable. Figure 1*D* illustrates the MSstatsTMT fit in the special case of protein MAP2K1 from Dataset 1, and supplemental Fig. S10*D* illustrates the same for protein MAP2K2 from Dataset 1. The fit for MSstatsTMT is a step function, and the figure highlights the presence of two biological replicates for each condition.

#### MSstatsTMT Null Hypothesis

Similar to SCAM, MSstatsTMT expresses the null hypothesis as a difference in means between treated vehicle is zero on average over the temperatures. Unlike SCAM, the flexible implementation of MSstatsTMT allows us to select a subset of the temperatures as shown in supplemental Table S2, Step 2, column four using custom contrasts. To avoid overfitting, the subset of the temperatures is chosen in advance, prior to analyzing the data. Figure 1, *D* and *H*, and supplemental Fig. S10, *D*–*H*, highlight the subset of temperatures used for a contrast with a blue box.

#### MSstatsTMT Model-based Inference

Model-based inference in MSstatsTMT relies on the standard likelihood-based procedure for contrast estimation in linear mixed-effects models (27, 28), as implemented in the R packages lme4 (43) and lmerTest (44) available on CRAN. When the contrasts involve a subset of the temperatures, the numerator of the test statistic (Step 5 in supplemental Table S2, column 4) is only calculated for the means in the subset, but the variance components are estimated from all the temperatures in the experiment, while accounting for the presence of two variance components. Under the null hypothesis, the test statistic approximately follows a Student distribution, where the degrees of freedom are estimated using the Sattherthwaite approximation (27, 28). The *p* values are adjusted for multiple testing with the method by Benjamini and Hochberg (40).

#### MSstatsTMT for Group Comparison Designs

MSstatsTMT is a general package for proteomic experiments with TMT labeling and multiple plexes. Beyond experiments with repeated measurements, the package is also applicable to experiments with group comparison designs. It is relevant for OnePot designs, where pooling all the temperatures into one channel reduces the experiment to a group comparison of conditions (such as treated *versus* control). Therefore, the null hypothesis is specified as a difference in the expected values between the conditions across the biological replicates. Experiments where all the biological replicates are allocated to the same plex have a single variance component, and model-based inference is simplified to Analysis of Variance (ANOVA). However, MSstatsTMT is also applicable to more complex group comparison designs, e.g., involving multiple drug concentrations or experiments where the biological replicates are allocated to multiple plexes, as described in the original MSstatsTMT publication (27).

## Experimental Procedures

We evaluated the impact of data processing and statistical modeling on the detection of protein interactions in thermal proteome profiling experiments as follows.

### Analysis of Thermal Proteome Profiling Experiments

The experimental datasets were prepared as described in the Datasets section and processed with both TPP and MSstatsTMT as described in supplemental Table S1. For each processing, the experimental and the simulated datasets were analyzed with TPP, NPARC, SCAM, and MSstatsTMT. For all the methods that report a single *p*-value (*i.e.*, all except TPP with the sigmoid model), protein interactions were determined according to the FDR-adjusted *p*-value cutoff of 0.05.

#### TPP Statistical Modeling

Following TPP processing, TPP statistical analysis was performed both as described in supplemental Table S2, column 1 (the spline fit) and supplemental Fig. S10, column 1 (the sigmoid fit). The MSstatsTMT processed input to TPP was unlogged and scaled as described in supplemental Table S1 Step 2, to make it compatible with the TPP modeling assumptions. For the spline version of TPP, the degrees of freedom of the splines were set to 5. For the sigmoid curve version of TPP, which outputs a separate *p*-value for each biological replicate, proteins were reported as interacting if: the *ΔT*_*m*_ between replicates had the same sign, and both replicates contained *p*-values <0.05, at least one replicate has R^2^ > 0.8, and all replicates lower sigmoid plateau <0.3.

#### NPARC Statistical Modeling

Following TPP processing, NPARC statistical analysis was performed as described in supplemental Table S2, column 2. The MSstatsTMT processed input to NPARC was unlogged and scaled as described in supplemental Table S1 Step 2, in the same way as for the TPP modeling. Post-processing filtering (supplemental Table S2, Step 4, column 2) was applied as recommended by the NPARC publication, where R^2^ > 0.8, and lower sigmoid plateau < 0.3^23^.

#### SCAM Statistical Modeling

Following TPP processing, the input to statistical modeling was the same as described for the TPP and NPARC statistical models. For MSstatsTMT processing, data were processed on the log scale as described in the MSstatsTMT workflow (27). SCAM was fit as described in supplemental Table S2, column 3. SCAM model parameters were set such that the fitted function remains monotonically decreasing, with *k* = 5 basis functions.

#### MSstatsTMT statistical Modeling

Following TPP processing, the input to statistical modeling was the same as described for the TPP and NPARC statistical models. Following MSstatsTMT processing, MSstatsTMT statistical analysis was performed as described in supplemental Table S2, column 4. After both processing types, the channel used for scaling or normalization was discarded and was not part of the statistical modeling. Since the experiments were expected to have overlapped vehicle and treated points at the very low and very high temperatures, the null hypothesis was stated in terms of a subset of temperatures t ∈ {6,7,8} for all the proteins as shown in supplemental Table S2, Step 2, column 4.

### Analysis of OnePot Experiment

The OnePot experiment (Dataset 4b) was processed as described in supplemental Table S1, except the spectrum-level normalization (Step 3) was set to global median normalization that equalizes the log_2_-transformed median intensities over all channels in the TMT-plex, all runs, and all features. The experiment was further analyzed with MSstatsTMT while accounting for the single-plex group comparison nature of the design. Protein interactions were determined by pairwise comparisons of every concentration to the vehicle condition.

## Results

### Data Processing and Statistical Modeling of Experimental Datasets Impact Curve Fitting for Known Interactors

Although we expected that multiple reasonable methods would provide similar conclusions, this was not the case in practice. For example, MAP2K1 (Fig. 1 and supplemental Fig. S9) and MAP2K2 (supplemental Figs. S10 and S11) are known to bind CH4987655 (Dataset 1). While processing made little difference for MAP2K2 (top and bottom rows of supplemental Figs. S10 and S11 have similar curves), processing had an impact on MAP2K1 as seen in Figure 1 and supplemental Fig. S9. For both MAP2K1 and MAP2K2, statistical models after the same processing produced different curve fits (panels of a same row show differently shaped curves). The difference in processing and/or in statistical modeling resulted in different *p*-values, and therefore in different conclusions regarding the presence of protein-drug interaction in some cases. For example, the combination of TPP processing and MSstatsTMT statistical modeling for protein MAP2K1 in Figure 1*D* was unable to detect the presence of this known interaction while MSstatsTMT processing and MSstatsTMT statistical modeling did present an interaction in Figure 1*H*. These discrepancies may be due to a combination of differences in data processing, and the lack of temperature coverage across the full range of unfolding and aggregation of this protein. Similar discrepancies resulting from both data processing and model fits were seen for other known protein interactions in Dataset 2 and Dataset 3a (supplemental Fig. S12–S25).

### Data Processing and Statistical Modeling of Experimental Datasets Impacts Overall Detection of Protein Interactions

**Fig. 2:**
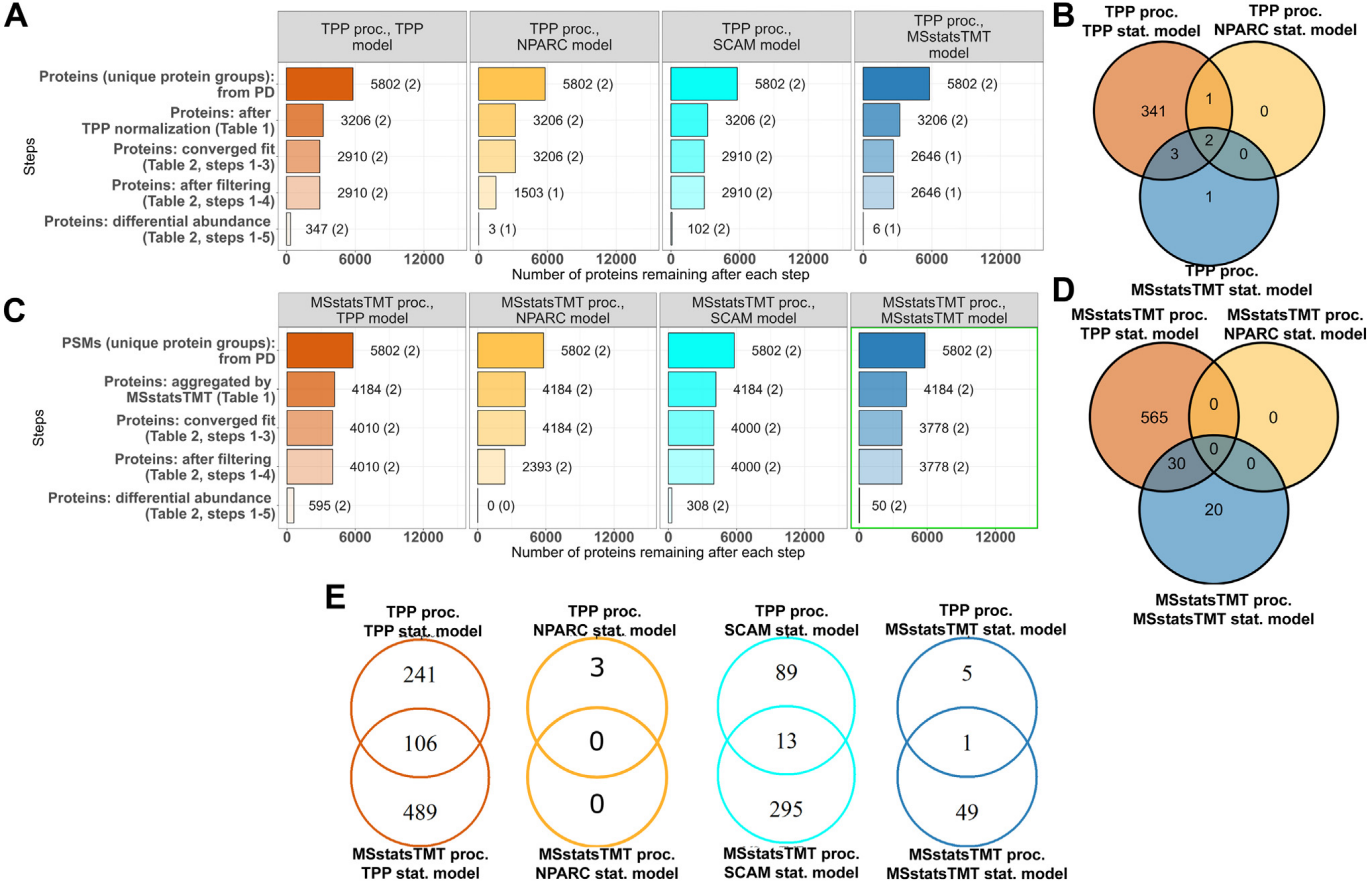
**Dataset 1- Phaneuf *et al,* detected protein interactions across all the proteins.***A*, TPP processing (supplemental Table S1, column 1; and vignette 1, section I). Each panel is a statistical model in supplemental Table S2. Steps are as in supplemental Table S2. The length of each bar and the number next to it indicate the number of proteins kept after that step. In parenthesis is the number of known protein interactions. *B*, TPP processing (supplemental Table S1, column 1; and vignette 1, section I). Overlap of detected protein interactions between statistical models in supplemental Table S2. *C*, as in (*A*), with MSstatsTMT processing (supplemental Table S1, column 2; and vignette 2, section I). *D*, as in (*B*), but with MSstatsTMT (supplemental Table S1, column 2; and vignette 2, section J). *E*, overlap of detected protein interactions between data processing choices, for same statistical models above. Despite detecting known protein interactions in most cases, the processing and statistical modeling steps detected different numbers of protein interactions, with a limited overlap.

We summarized the number of protein interactors detected over all the choices of data processing and statistical modeling in Dataset 1 (Fig. 2). Figure 2*A* compares the detection of protein interactions after the TPP processing, according to the different statistical models. All the statistical modeling approaches took as input the same 3206 proteins; however, all but MSstatsTMT lost one interactor at the model fitting step due to MAP2K1 having a single measure per condition. NPARC lost proteins after the model fit, due to post-processing filtering. The last model-based inference step produced additional discrepancies. As a result, the statistical models detected a broadly different number of protein interactions, ranging from 347 to 3. Although all but NPARC and MSstatsTMT detected both known protein interactions after the TPP processing, the overlap for the interacting proteins highlights two interactions (Fig. 2*B*). NPARC and MSstatsTMT confirmed one of the two known interactions of the compound.

**Fig. 3:**
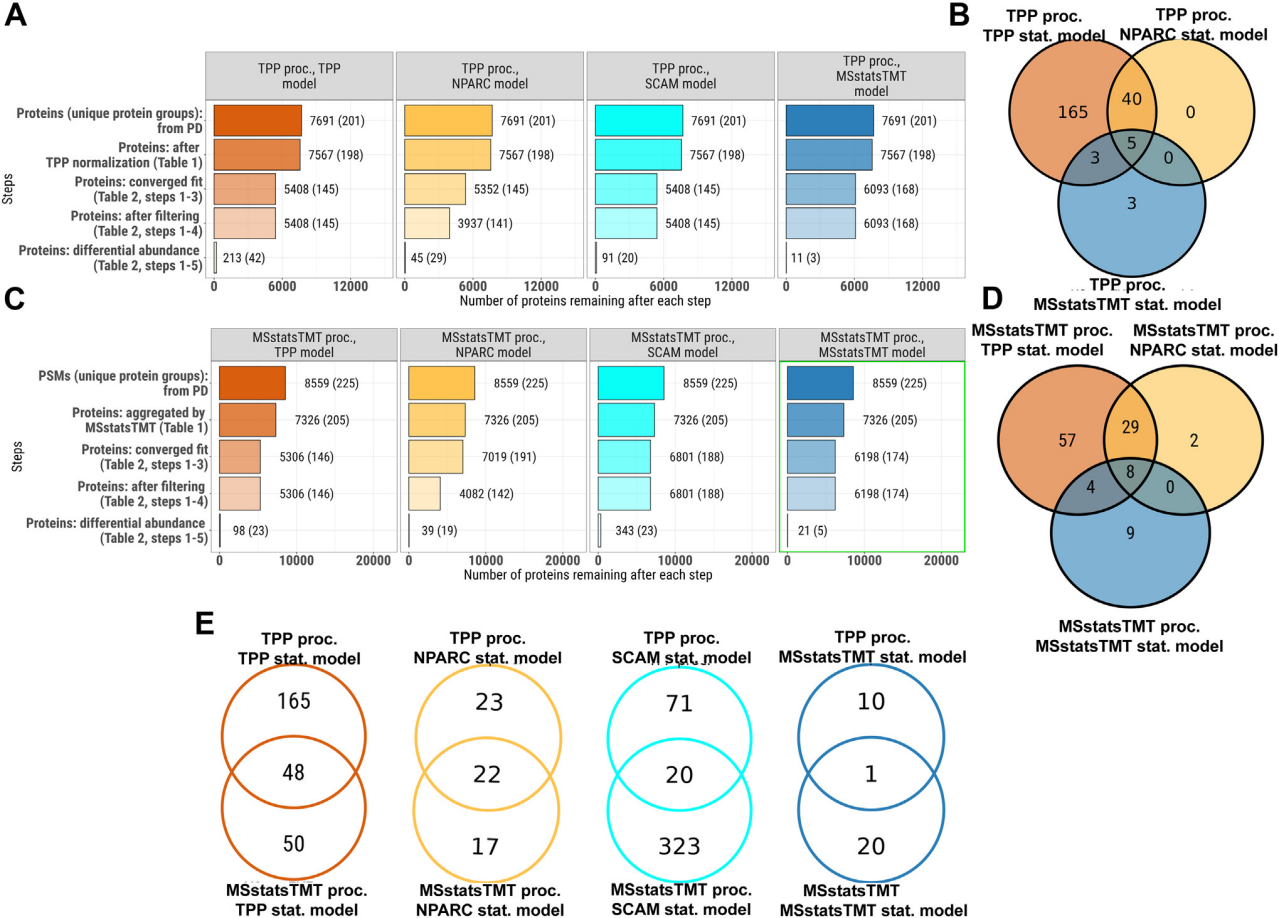
**Dataset 3a thermal profile - Xu *et al*, detected protein interactions across all the proteins.** All panels are as in Figure 2, following supplemental Table S1, column 1; and vignette 5, section J for panels (*A* and *B*); and supplemental Table S1, column 2; and vignette 6, section J for panels (*C* and *D*). *E*, the processing and statistical modeling steps detected different numbers of protein interactions, different number of known targets (out of 237 total), with limited overlap.

We next compared the identification of protein interactions using different statistical models after processing with MSstatsTMT (Fig. 2, *C* and *D*). The MSstatsTMT input had a larger number of proteins, due to its less stringent input-level filtering criteria. Working with the input data at the MS feature level, MSstatsTMT processing started with 5802 protein groups. However, MSstatsTMT converters filtered out some protein groups as described in supplemental Table S1. After protein-level quantification, only 4184 proteins were available for statistical modeling. As above, all the models but NPARC lost proteins at the model fitting step, and NPARC lost proteins at the post-processing filtering step. Overall, all the methods except NPARC detected more protein interactions, including the two known interactions for the compound. NPARC was unable to detect the two known interactions at the feature-level in Figure 2*C* because shared protein groups were filtered out. Therefore, the overlaps between TPP in Figure 2*D* show more overlaps between TPP and NPARC. Finally, Figure 2*E* shows that the choice of data processing plays an important role in the detection of protein interactions, as even the results of the same statistical model after different processing had little overlap.

supplemental Fig. S24 repeats the trends of Figure 2, with TPP model using the sigmoid fit. Figure 3, as well as supplemental Fig. S25 show the same analyses of Dataset 3, and report a similar extent of discrepancies between choices of data processing and statistical modeling. supplemental Figs. S26 and S27 reproduce the analyses in Figures 2 and 3, but using different subsets of temperatures for the MSstatsTMT model, and illustrate that the choice of the temperature subset also affects the results.

Overall, the results point to poor reproducibility of the detection of protein interactions across evaluated choices of data processing and statistical models. Although the details were dataset-specific, some patterns emerged. In most cases, the TPP model with splines was among the most sensitive, followed by SCAM. The NPARC statistical model had the highest variability in the number of detected protein interactions between the processing choices and between the datasets. MSstatsTMT was consistently reporting the smallest number of detected protein interactions, especially when the subset of temperatures was close to the upper or lower plateau. The lack of sensitivity by MSstatsTMT is not surprising, since its statistical model must estimate the largest number of parameters and components of variation.

### TPP with splines, NPARC, and SCAM produced false positive and false negative results, while MSstatsTMT was well calibrated in simulated data

**Fig. 4:**
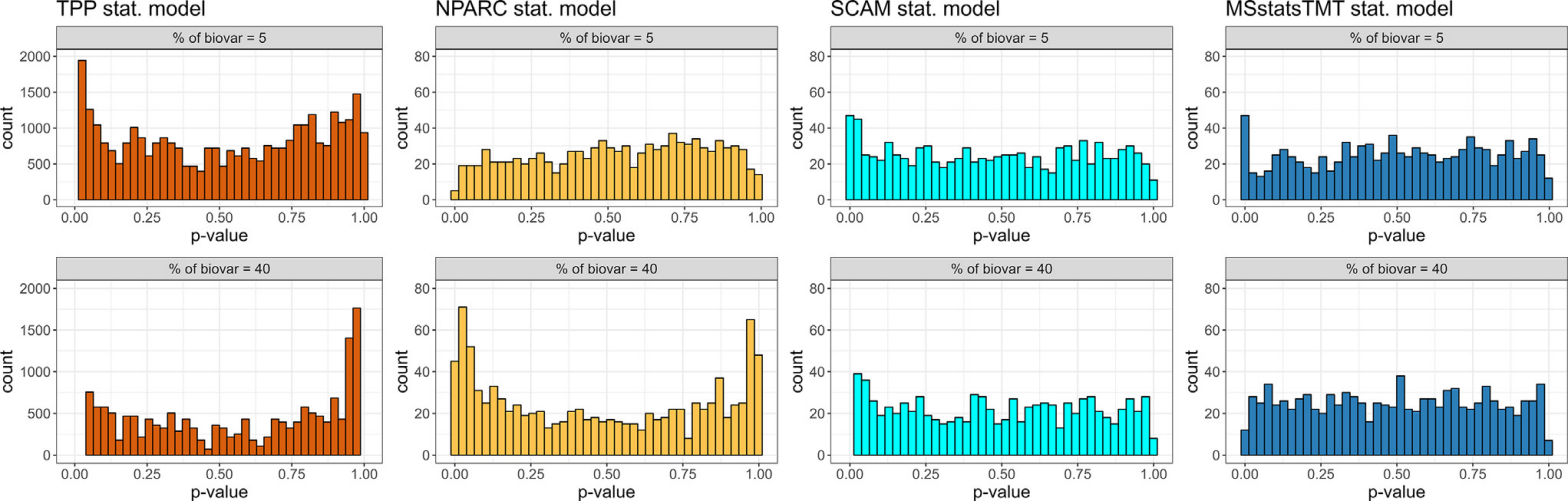
**Simulated dataset, specificity of statistical models in the case of a non-interacting protein (vignette 9).** The simulation mimicked a non-interacting protein, using as a template the thermal profile of protein Stat3 from Dataset 2, processed with MSstatsTMT (supplemental Table S1, column 2). Columns: statistical models in supplemental Table S2. Rows: ICC, *i.e.* percentages of biological between-replicate variation in the total biological and technological variation for that protein. Each cell is a histogram of *p*-values of 1000 instances of simulating the protein at the specific biological variation. The *p*-values of a well-calibrated statistical model are expected to be uniformly distributed. Concentrations of low *p*-values indicate overfitting. Concentrations of large *p*-values indicate under-fitting. MSstatsTMT was most calibrated. TPP and SCAM were most prone to over- or under-fitting.

Since the statistical models varied in performance, we set out to assess whether more sensitive models reported more true protein interactions. Since the ground truth for all the proteins in the experimental datasets was unknown, we investigated this by simulating 1000 proteins with no drug interaction. The simulation was inspired by the experimental datasets, as described in Section Experimental Datasets. When a protein does not interact with the drug, the null hypothesis is true, and the *p*-values of hypothesis tests are expected to be uniformly distributed. Concentrations of *p*-values close to 0 indicate overfitting, and concentrations of *p*-values close to one indicate underfitting. Figure 4 summarizes the outcomes of simulations for all the statistical models producing a single *p*-value (*i.e.*, all except TPP with sigmoid fit), for three different levels of biological variation. The figure illustrates that model-based inference in TPP with splines, NPARC, and SCAM was poorly calibrated. In other words, the models tended to overfit (*i.e.*, produce more false positives than was expected by random chance), and in the case of TPP and NPARC, also underfit (*i.e.*, produce more false negatives than was expected by random chance), regardless of the extent of biological variation. The model-based inference by MSstatsTMT was the only well-calibrated approach.

### In simulations, trading off temperatures for biological replicates increased the sensitivity of MSstatsTMT, without compromising the specificity or the experimental cost

**Fig. 5:**
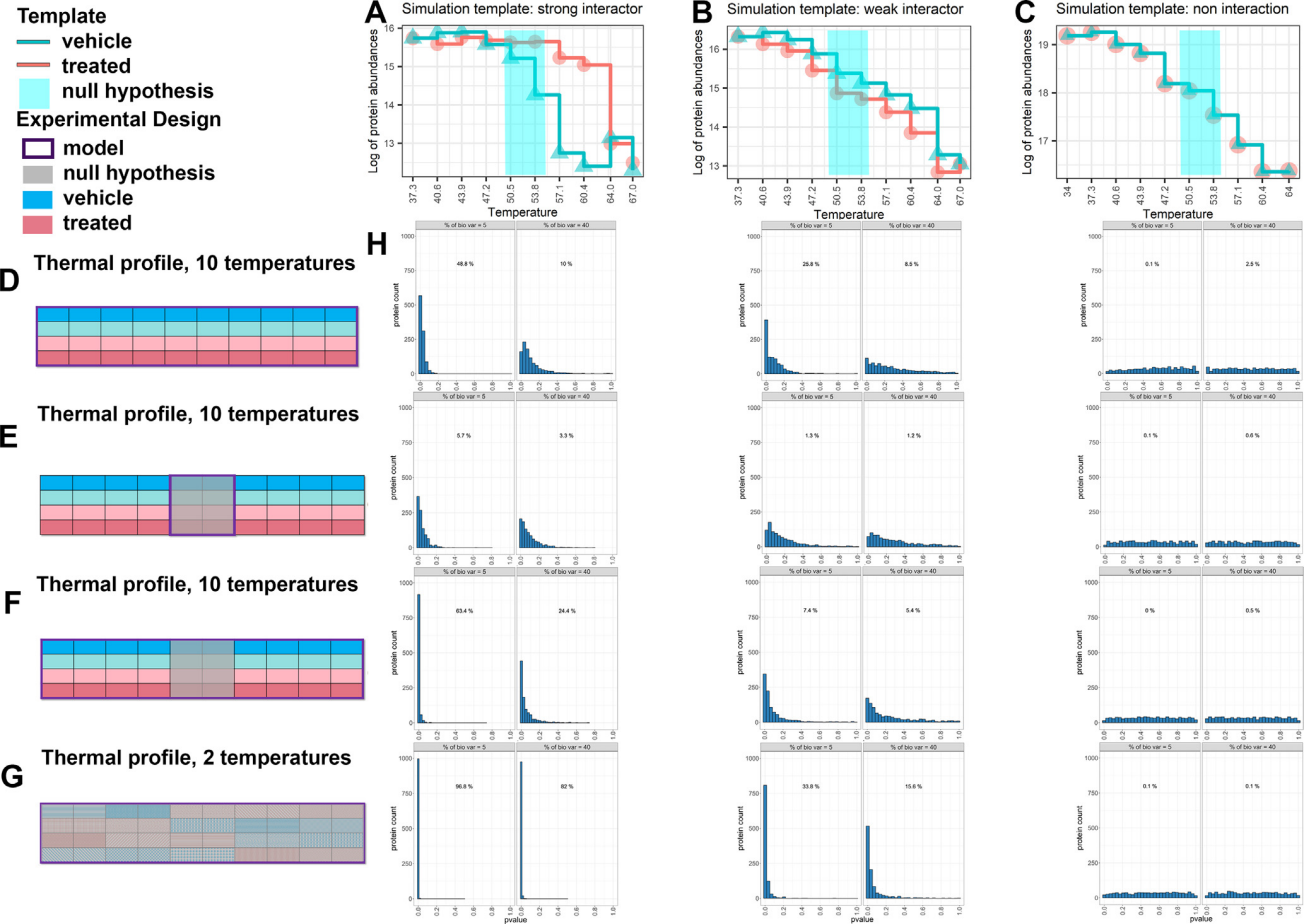
**Simulated dataset, sensitivity and specificity of MSstatsTMT for thermal profiling experiments with different designs (vignette 10).***A–C*, ground truth templates for the simulation. Labels and colors are as in Figure 1. *A*: MSstatsTMT fit to a strong interacting protein MAP2K2 in Dataset 1, *B*: MSstatsTMT fit to a weak interacting protein SAP18 in Dataset 1, *C*: MSstatsTMT null hypothesis fit for a non-interacting protein STAT3 in Dataset 2. *D–G*, experimental designs for each simulation. *D*, Two biological replicates per condition, 10 temperatures per replicate are used to both fit the model and specify the null hypothesis. Each biological replicate is allocated to a separate plex. *E*, only two temperatures are used for both statistical modeling (*purple box* in figure, Step 1, column four in supplemental Table S2) and hypothesis testing (*grey area* in figure, Step 2 of supplemental Table S2, column 4). These temperatures are also highlighted by grey areas in panels *A–C*. *F*, as in (*D*); however, while all the temperatures are used for statistical modeling (Step 1 in supplemental Table S2, column 4), only two temperatures are used for hypothesis testing (Step 2 in supplemental Table S2, column 4). This approach was used for the analyses of the experimental datasets in this manuscript. *G*: Ten biological replicates per condition, two temperatures per replicate (*grey areas* in panels *A–C*), randomly allocated to four plexes. *H*, each subpanel is a histogram of *p*-values of 1000 simulated instances from the templates above, for each experimental design, with biological between-replicate variation representing 5% or 40% of the total biological and technological variation. Percentage in the cell marks the proportion of *p*-values below the 0.05 cutoff. Design in (*G*), which traded off temperatures for an increased number of biological replicates, maximized the sensitivity of MSstatsTMT-based detection of both strong and weak protein interactions, without compromising the specificity of the results.

Since in the results mentioned earlier established that MSstatsTMT was best-calibrated statistical model but lacked sensitivity, we sought ways to increase the sensitivity of MSstatsTMT without compromising the specificity and without increasing the total experimental cost. To evaluate both the sensitivity and the specificity of the approaches, we performed simulations described in the Experimental Datasets section, with the results summarized in Figure 5. The simulations mimicked a strong protein interaction (Fig. 5*A*), a weak protein interaction (Fig. 5*B*) and an absent protein interaction (Fig. 5*C*). We considered an experimental design with two biological replicates per condition and 10 temperatures, with all the data available for both model fitting and hypothesis testing (Fig. 5*D*). We compared this design and analysis approach to three alternatives. First, since MSstatsTMT does not fit a parametric curve, we can reduce the total number of temperatures. Therefore, we reduced the number of temperatures to two in both model fitting (Step 1 of supplemental Table S2, column 4) and hypothesis testing (Step 2 of supplemental Table S2, column 4), as shown in Figure 5*E*, and discarded the remaining channels. Second, we used all the 10 temperatures to fit the model (Step 1 of supplemental Table S2, column 4) but reduced the number of temperatures in the null hypothesis to two as described in Step 2 of supplemental Table S2, column 4 and shown in Figure 5*F*. This was the MSstatsTMT analysis of the experimental data in this manuscript. Finally, we returned to the experimental design in Figure 5*E*, but used the channels freed by the smaller number of temperatures to increase the number of biological replicates to 10 per condition as shown in Figure 5*G*.

Figure 5*H* summarizes the results of the simulations. Columns of the panels match the simulation templates. Rows of the panel match the experimental designs in Figure 5, *D*–*G*. The two subpanels represent two extents of between-replicate biological variation, namely 5% and 40%. We can make several conclusions. First, simulations of the design in Figure 5*D* confirmed the low sensitivity of MSstatsTMT with all the temperatures for even strong protein interactions. However, removing the remaining temperatures from the model fit as in Figure 5*E* was counterproductive. Indeed, this design lost valuable degrees of freedom, and the estimation of variation became less precise. Third, reducing the number of temperatures as shown in Figure 5*F* helped increase sensitivity because the null hypothesis did not include the temperatures where the curves were likely to overlap. Finally, the design in Figure 5*G*, maximizing the number of biological replicates with two temperatures each, was the most principled approach to increase the sensitivity for both strong and weak interactions, without compromising the specificity of the results, and without increasing the experimental cost.

### Simulations show that pooling multiple temperatures and allocating them to a single channel in a OnePot design increased the sensitivity of MSstatsTMT, without compromising the specificity or the experimental cost

**Fig. 6:**
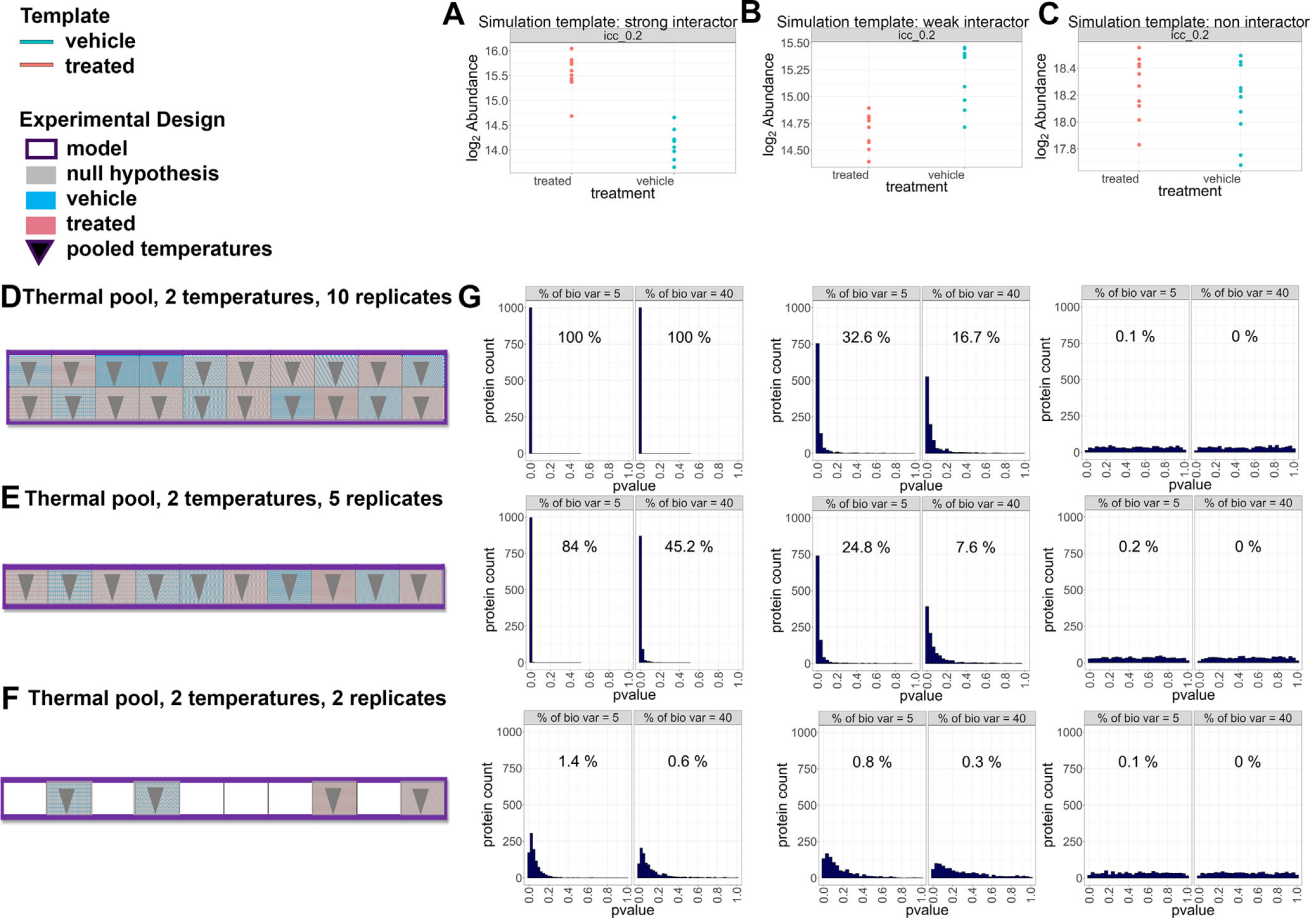
**Simulated dataset, sensitivity and specificity of MSstatsTMT for OnePot experiments with different designs (vignette 11).***A–C*, one simulation instance of a pooled dataset for strong, weak and non-protein interactions. The pools used the templates and the subset of temperatures in Figure 5, *A–C*. *D–F*, experimental designs for each simulation. *D*, ten biological replicates per condition, each containing a pool of two temperatures. The replicates were randomly allocated to two plexes. *E*, As in (*D*) but with five biological replicates per condition, allocated to one plex. *F*, as in (*D*) but with two biological replicates per condition and one (incomplete) plex. *H*, panels and labels as in Figure 5*H*. The pooled designs in 6D increased the sensitivity of detecting protein interactions without compromising the specificity, and controlled the experimental cost, as compared to the designs in Figure 5.

Encouraged by the positive impact of increasing the number of biological replicates, we considered taking the design in Figure 5*G* further by pooling multiple temperatures, allocating them to a single channel, and dedicating the remaining channels to more biological replicates. To contrast such OnePot experiments to the thermal profiling experiments above, we simulated pooled versions of the same strong, weak, and non-interacting templates as in Figure 5, *A*–*C*, as described in the Experimental Datasets section. The simulation pooled the subsets of temperatures highlighted by the grey areas in Figure 5, *A*–*C*. Figure 6, *A*–*C* illustrates one randomly chosen instance of these simulated datasets. We evaluated a OnePot design that used the same number of biological replicates (10 per condition) as in Figure 5*G*, shown in Figure 6*D*. We further considered a OnePot design that reduced the number of biological replicates to five per condition, such that the entire experiment could be performed in a single plex (Fig. 6*E*). Finally, similarly to the thermal profiling experiments, we considered two biological replicates per condition, in a way that left some channels unused (Fig. 6*F*). Figure 6*G* shows that in these simulations, OnePot experiments with 10 biological replicates exceeded the sensitivity of any thermal profiling designs in Figure 5, without compromising the specificity of the results. The experiments could tolerate a reduction of the number of biological replicates to five per condition. Reducing the number of pooled replicates to two per condition, as in thermal profiling experiments, reduced the sensitivity, especially for the weak interaction.

### In experimental datasets, OnePot experiment with multiple drug doses, followed by MSstatsTMT modeling, increased the sensitivity of detecting protein interactions

To further evaluate the properties of OnePot designs in real-life experiments, we compared the detection of protein interactions in thermal proteome profiling experiment in Dataset 3a to that of the OnePot experiment in Dataset 3b. For Dataset 3a we considered the two choices of data processing in supplemental Table S1, and all the statistical models in supplemental Table S2. Dataset 3b had a one-plex group comparison OnePot design with more biological replicates per condition and incorporated additional replication in form of multiple drug concentrations. Since the TPP processing and modeling was not applicable to this dataset, we considered protein-level summarization by Proteome Discoverer and by MSstatsTMT, followed by the MSstatsTMT statistical model. We compared protein interactions detected by these analyses to the protein interactions in the reference set, described in the Experimental Datasets section.

**Fig. 7:**
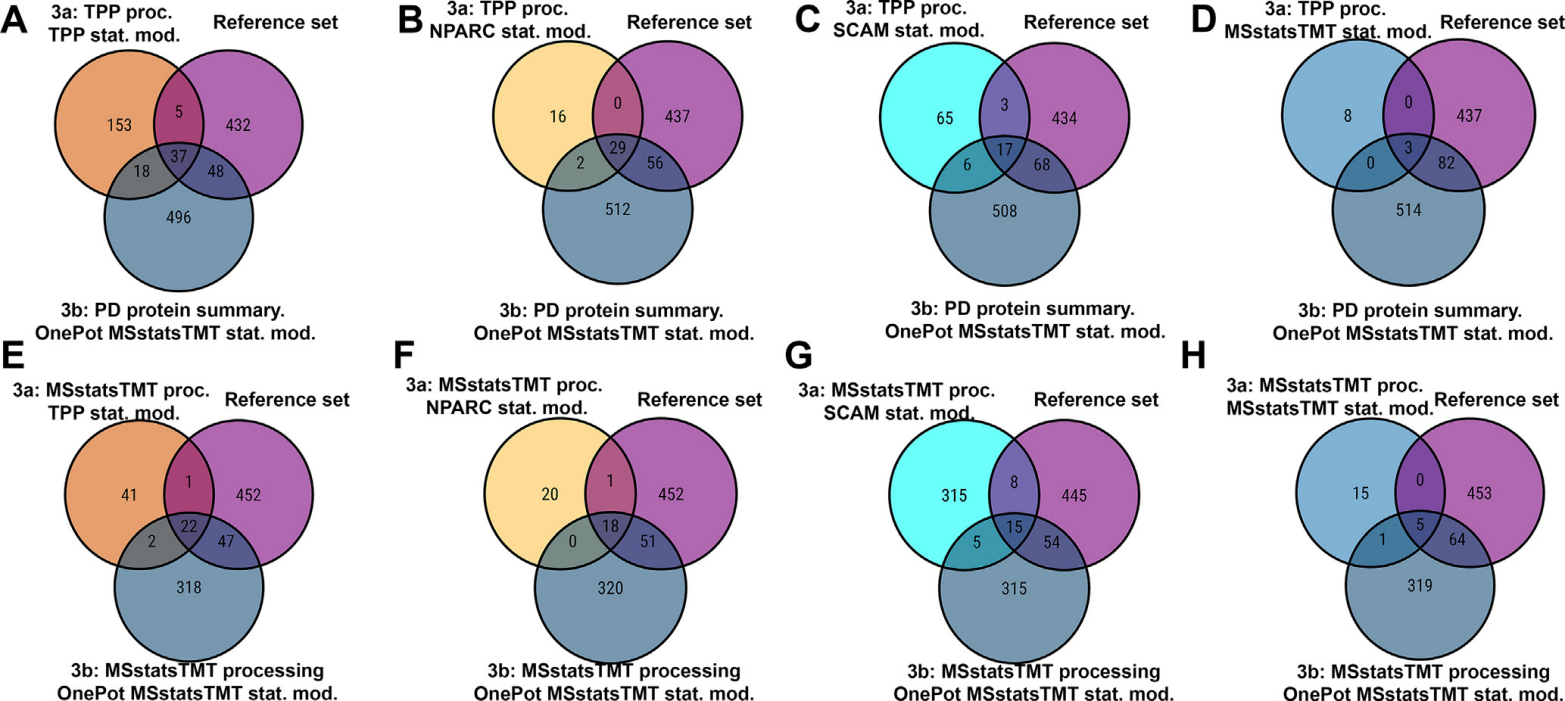
**Datasets 3a-thermal and 3b-OnePot - Xu *et al.*, overlap of detected protein interactions between the two experiments, and their agreement with the reference set of protein interactions, for each data processing and for each statistical model in**supplemental Table S2. *A–D*: the thermal profiling experiment was processed with TPP, and OnePot experiment was summarized at the protein level by Proteome Discoverer (vignettes five and 7). *E–H*, both thermal profiling experiment and OnePot experiment were processed with MSstatsTMT (vignettes six and 8). The OnePot experiment detected more interacting proteins from the reference set than any of the thermal profiling experiments.

Figure 7 summarizes the detection of interacting proteins for all the analyses. Similarly to the simulation-based results, the experiment with OnePot design and more replicates had a higher sensitivity and a higher overlap with the reference set than the thermal profiling experiment. This trend held for all the processing and all the statistical modeling strategies. The number of protein interactions detected in the OnePot experiment after MSstatsTMT processing was smaller than after the Proteome Discoverer protein-level summarization. This was primarily due to the removal of shared protein groups and the merging of protein isoforms by MSstatsTMT.

### In experimental datasets, the OnePot experiment with multiple drug doses, followed by MSstatsTMT modeling, increased the reproducibility of MSstatsTMT across choices of data processing

**Fig. 8:**
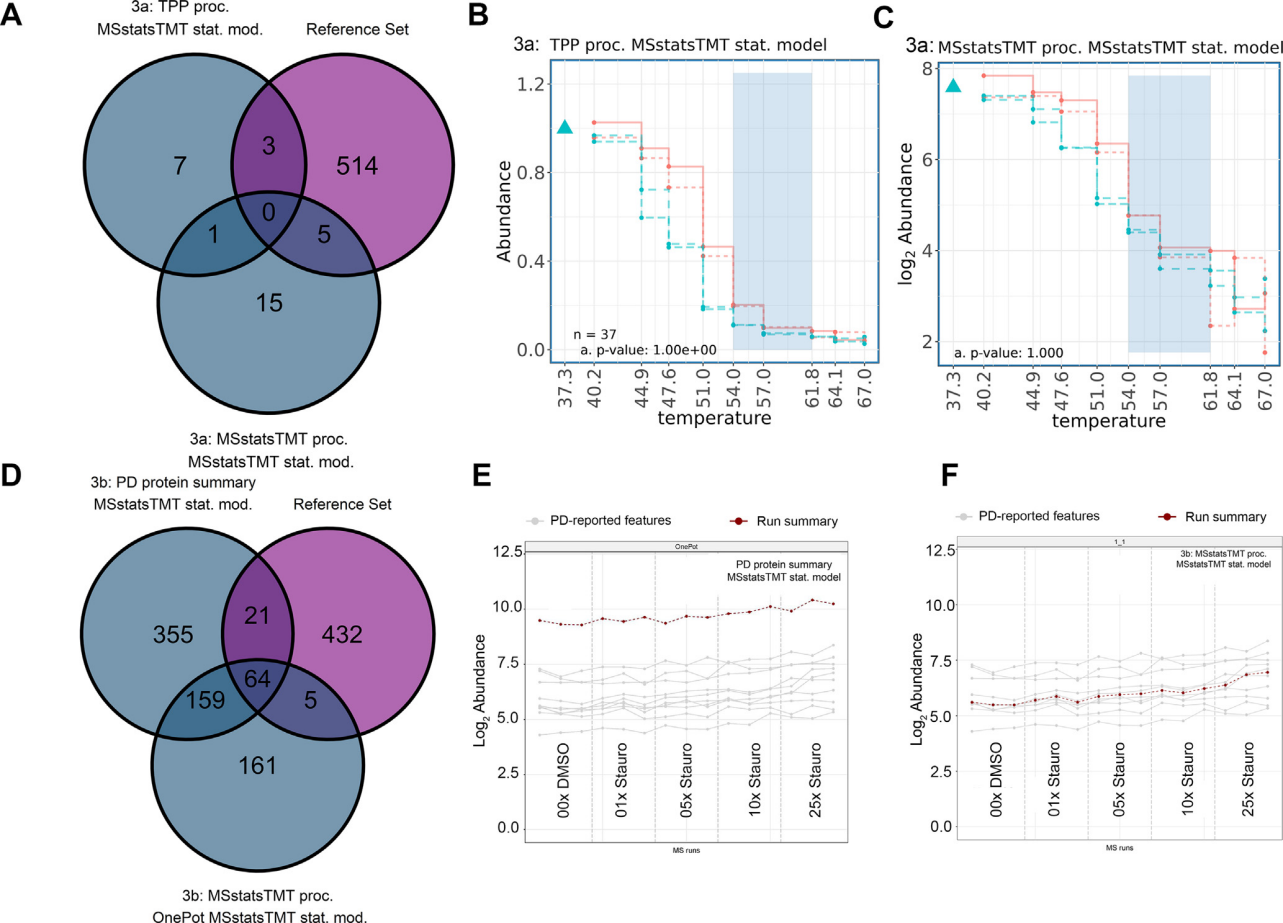
**Datasets 3a-thermal and 3b-OnePot - Xu *et al*., overlap of protein interactions detected with different choices of data processing, modeled by MSstatsTMT, and their agreement with the reference set of protein interactions.***A–C*, thermal profiling experiment, Dataset 3a (vignettes five and 6). *A*, overlap between protein interactions detected after TPP processing and after MSstatsTMT processing. *B*, profile plots for MAP2K2 in Dataset 3a, after TPP processing. *C*, as in (*B*), but after MSstatsTMT processing. *D*–*F*: OnePot experiment, Dataset 3b (vignettes seven and 8). *D*, overlap between protein interactions detected after protein-level summarization by Proteome Discoverer and after MSstatsTMT processing. *E*, profile plots for MAP2K2 in Dataset 3b, after protein-level summarization by Proteome Discoverer. *F*, same as (*E*) but after MSstatsTMT processing. The OnePot experiment demonstrated greater sensitivity and greater robustness to the choices of data processing than the thermal profiling experiment.

Finally, we compared the thermal proteome profiling experiment in Dataset 3a to that of the OnePot experiment in Dataset 3b in terms of robustness of detected interactions to choices of data processing. As above, we evaluated the results in the context of the reference set of protein interactions described in the Experimental Datasets section. For Dataset 3a, the comparison considered both choices of data processing in supplemental Table S1, followed by the MSstatsTMT model. For Dataset 3b, we considered protein-level summarization by Proteome Discoverer reported by Xu *et al.* (18), as well as MSstatsTMT processing that started from PSM files reported by Proteome Discoverer, followed by the MSstatsTMT model. Figure 8*A* shows that the thermal profiling experiment in Dataset 3a had almost no overlap between protein interactions detected after TPP processing and after MSstatsTMT processing, and both methods had almost no overlap with the reference set. Figure 8, *B* and *C* shows the profiles of the known interactor MAP2K2 for the two choices of data processing (TPP and MSstatsTMT, respectively), and illustrates the fact that the data processing of thermal profiling experiments can substantially impact the conclusions. Figure 8*D* illustrates that, although the OnePot experiment was also affected by data processing, it was more robust, and more protein interactions were found with both processing choices. Figure 8, *E* and *F* show the effect of processing affects the dose–response profile of MAP2K2. As can be seen, differences between the control (DMSO) and drug treatment were more apparent in the OnePot experiments than in the thermal profiling experiments, due to both an increase in replication and to presence of multiple concentrations. supplemental Fig. S28 provides an additional insight into the summarized abundances of MAP2K2, another known interaction CHEK1, as well as not a known interaction protein STK2. The figure further illustrates that, while data processing affects the details of dose-dependent patterns of protein abundances, the replication and the presence of multiple concentrations helped detect the interaction.

## Discussion

Experimental design, data processing, and statistical analysis play a key role in thermal proteome profiling, and multiple choices are currently available. The analyses of experimental thermal profiling datasets in this manuscript painted a bleak picture regarding the reproducibility of the results across choices of data processing and statistical modeling. The observed discrepancies were due to multiple issues, including subjective choices of data processing and filtering, restrictive modeling assumptions, failure of some of the models to account for all the sources of between-replicate and within-replicate variation. However, most importantly, the discrepancies were due to underpowered experimental designs caused by practical or financial reasons. These deficiencies manifested themselves in over- or under-fitting, and lack of reproducibility of detecting protein interactions.

This manuscript demonstrated that this situation can be generally improved by considering experimental designs that either reduce or pool together some of the temperatures and use the freed experimental resources to increase the number of biological replicates. These new designs need to be accompanied by statistical methods that do not rely on complex curve fitting, can flexibly accommodate a smaller number of temperatures and a larger number of biological replicates, and appropriately describe all the sources of variation, including from multiple drug concentrations. MSstatsTMT offers such a general statistical modeling framework. We therefore advocate for the introduction of such designs, followed by analyses by MSstatsTMT.

The recommendation comes with some caveats. For example, the manuscript demonstrated the importance of choosing the right subset of temperatures for stating the null hypothesis with MSstatsTMT. It is important that the subset of temperatures is selected before collecting the data and starting the analysis, to avoid overfitting. This can be implemented by leveraging prior studies with same or similar drugs. Moreover, any data processing step, including by MSstatsTMT, unavoidably makes subjective decisions regarding issues such as filtering of PSMs or proteins, treatment of missing values, protein-level summarization or normalization, and more work in these areas is needed. Despite the challenges, we believe that the proposed approach is ready for practical applications and can be a great help in target validation and drug development. We have outlined the recommended Thermal and OnePot designs in the vignettes under the recommended workflow folder in the repository.

## Data Availability

Dataset raw mass spectrometry data files were downloaded and available through the ProteomeXchange Consortium *via* the PRIDE partner repository. Dataset 1: Mass spectra from the original publication were downloaded from the PRIDE under accession PXD034087. We re-processed these files in Proteome Discoverer and listed the search settings in the Data processing section as shown in Supplemental Data. Vignettes describing all the analysis steps are available at MassIVE.quant under MassIVE ID MSV000092551. Dataset 2: Mass spectra from the original publication were downloaded from PRIDE under accession PXD017419 (11). We re-processed these files in Proteome Discoverer (version 2.5.004) and uploaded these to MassIVE.quant under MassIVE ID MSV000088522. Users can access the reanalysis file with identifier RMSV000000695.1. Step-by-step R vignettes are included here (https://github.com/Cleanvalidation/MSstatsThermalProfile/tree/main/vignettes).

## Supplemental data

This article contains supplemental data (5, 18, 25, 33, 37, 45).

## Conflict of interest

The authors declare that they have no conflicts of interest with the contents of this article.
