# Supplementary material for: MSstatsTMT Improves Accuracy of Thermal Proteome Profiling
Source: Mol Cell Proteomics. 2025 May 27;24(8):100999. doi: 10.1016/j.mcpro.2025.100999 (PMC12335990; doi:10.1016/j.mcpro.2025.100999)
Supplement: Supplementary Material [file mmc1.pdf]

MSstatsTMT improves accuracy of thermal proteome profiling by trading off temperatures and biological replicates

Amanda M. Figueroa-Navedo[1], Rohan Kapre[2], Tushita Gupta[2], Yingrong Xu[3], Clifford G. Phaneuf[1,4], Pierre M. Jean Beltran[5], Liang Xue[5], Alexander R. Ivanov[1] and Olga Vitek*[2]

Supplemental materials

[1]Barnett Institute of Chemical and Biological Analysis, Department of Chemistry and Chemical Biology, Northeastern University, Boston, MA, US

[2]Khoury College of Computer Science, Northeastern University, Boston, MA, US

[3]Discovery Sciences, Pfizer Inc., Groton, CT, US

[4]Sanofi, Disease Profiling and Functional Genomics, Cambridge, MA, USA

[5]Machine Learning and Computational Sciences, Pfizer Inc., Cambridge, MA, US

Contents

1. **Experimental datasets** **S-1**
2. **Background**
   2.1 Data structures and notation **Table S-1**
   2.2 Data processing methods for thermal proteome profiling **S-7**
   2.3 Statistical models for thermal proteome profiling **Table S-2**
3. **Evaluation: Detection of protein interactors was impacted by both data processing method and choice of statistical model** **S-23**

1. Experimental datasets

Data processing

Dataset 1-Phaneuf *et. al*

The thermal profiles were processed with Proteome Discoverer (PD) version 2.4 by the original authors [27] We exported PSM and protein reports for Carrier-FAIMS-eFT from the result files. The PSM reports contained PSM mapped to unique and shared protein groups. We filtered out PSM with shared protein group membership, and only retained unique protein groups to avoid signal contributions from other proteins. In the protein reports built by Proteome Discoverer from the filtered PSM files, 44.7% of the unique protein groups had more than three missing values in at least one plex (Supplemental Fig S-1), and these protein groups were removed from consideration. We preserved the filtered PSM-level and protein-level files for further analyses and evaluation.

Dataset 2- Leijten *et. al*

While the original publication quantified proteins with MaxQuant, in this manuscript we reprocessed the data with Proteome Discoverer (version 2.5.004). Raw data were downloaded from PRIDE under accession PXD017419 and then processed using Proteome Discoverer with the SEQUEST HT search engine, and TrEMBL Zebrafish database downloaded on 11-17-2021. The Proteome Discoverer workflow mirrored the parameters outlined in the original publication for MaxQuant. We exported PSM and protein reports from the result files. The PSM reports contained PSM mapped to unique and shared protein groups. We filtered out PSM with shared protein group membership, and retained unique protein groups. In the protein reports built by Proteome Discoverer from filtered PSM files, only 0.37% of the proteins had more than three missing values in at least one plex (Supplemental Fig S-2). Therefore, no additional proteins were filtered out. We preserved the filtered PSM-level and protein-level files for further analyses and evaluation.

Dataset 3a-Xu *et. al*

The authors of the original publication processed the raw data with Proteome Discoverer (version 2.4). Since protein abundance at 75°C was low, the original publication by Xu *et al.* discarded this channel. Therefore, this channel was also discarded in this manuscript. MSF files from the original publication were combined into a single multi-consensus Proteome Discoverer workflow. We extracted PSM and protein reports, with the following relevant settings: site probability threshold of 75, co-isolation threshold of 50%; signal-to-noise threshold of 10, 1% feature and peptide FDR[33]. In the PSM report, we filtered out PSM with shared protein group membership, and retained unique protein groups. Isoforms of the same peptide sequence were mapped to the same unique protein sequence. In the protein reports built by Proteome Discoverer from the filtered PSM files, none of the proteins had more than three missing values in at least one plex (Supplemental Fig S-3), and no filtering was necessary. We preserved the filtered PSM-level and protein-level files for further analyses and evaluation.

Dataset 3b OnePot- Xu *et. al*

As in Dataset 3a, the authors of the original publication processed the raw data with Proteome Discoverer (version 2.4). Both PSM reports and protein-level summary reports were exported from the Proteome Discoverer result files produced by the original publication. In the protein reports built by Proteome Discoverer from filtered PSM files, no further filtering was necessary at the protein level.

Column		1	2
**Step**	**Workflow**	**NPARC (version 1.10.0) & TPP (version 3.26.0)**	**MSstatsTMT (proposed, version 2.6.0)**
1	**Read**	$Y^{PD}_{pcrt},$ $p=1,\ldots,P$, $c=0,1$, $r=1,\ldots,R$, $t=0,\ldots,T$	PSM intensities $X^{PD}_{pcrti}, p=1,\ldots,P, c=0,1, r=1,\ldots,R, t=0,\ldots,T, i=1,\ldots,I$
2	**Reporter ion abundance transformation**	$Y'_{pcrt}=Y^{PD}_{pcrt}/Y_{pcr0}$ *	$X'_{pcrti}=log_2(X^{PD}_{pcrti})$ **
3	**Filter out from each c and r**	none	shared peptides, PSMs with ≥9 missing values, ** proteins with one feature
4	**Spectrum level normalization**	none	set to FALSE **
5	**Protein summarization**	none	$Y'_{pcrt}=Tukey\ median\ polish\ with\ imputation\ (X'_{pcrti})$
6	**Protein normalization**	Normalized w.r.t. factor Q_T as calculated from a subset of proteins as described in Supplemental Fig.(S-8) $Y_{pcrt}=Y'_{pcrt} * Q_t$	$Y_{pcrt}=equalize\ log_2\ intensities\ (Y'_{pcr0})$ apply same shifts to other channels

Table 1. **Steps of data processing, prior to statistical modeling.** NPARC recommends processing with TPP. Specifically, TPP takes as input protein-level reporter ion abundances summarized, e.g., by Proteome Discoverer (Supplemental Table 1, Step 1). Next, the abundances are ratioed over the reference channel Y_{pcr0} (Supplemental Table 1, step 2) and normalized as outlined in Algorithm 3 in Supplemental Fig S-7. * indicates a step that requires manual conversion of the data to Bioconductor's ExpressionSet class by the user. In contrast, MSstatsTMT takes as input peptide-spectral matches (called features) from a data processing tool (Supplemental Table 1, step 1). Next, an MSstatsTMT converter performs operations such as log_2 reporter ion abundance transformation (Supplemental Table 1, step 2), and filtering of peptides shared between proteins, PSM plexes with nine or more missing values, and proteins identified by only one feature (Supplemental Table 1, step 3). ** indicates that these steps are fully automated by the converter. Supplemental Table 1, step 5 of MSstatsTMT summarizes the protein-specific features into a single protein abundance. Supplemental Table 1, step 6 of MSstatsTMT equalizes log_2 intensities for the reference channel (Y'_{pcr0}).

Step	Statistical model	TPP (version 3.26.0)	NPARC (version 1.10.1)	SCAM splines (version 1.2-13)	MSstatsTMT (version 2.6.0)
1	**Model**	$Y_{crt} = f_c(t) + \epsilon_{crt}$<br>$f_c(t) = \beta_{0c} + \sum_b s_{bc}(t)$<br>$0 \leq t \leq T$<br>$\epsilon_{crt} \overset{\text{i.i.d.}}{\sim} N(0, \sigma^2)$	$Y_{crt} = f_c(t) + \epsilon_{crt}$<br>$f_c(t) = \frac{1 - p_c}{1 + e^{-\left(\frac{a_c}{t} - b_c\right)}} + p_c$<br>$0 \leq t \leq T$<br>$\epsilon_{crt} \overset{\text{i.i.d.}}{\sim} N(0, \sigma^2)$	$Y_{crt} = f_c(t) + u_{cr} + \epsilon_{crt}$<br>$f_c(t) = \beta_{0c} + \sum_b s_{bc}(t), \frac{df_c(t)}{dt} \leq 0$<br>$0 \leq t \leq T$<br>$u_{cr} \overset{\text{i.i.d.}}{\sim} N(0, \sigma_u^2), \epsilon_{crt} \overset{\text{i.i.d.}}{\sim} N(0, \sigma^2)$	$Y_{crt} = \mu_{ct} + u_{cr} + \epsilon_{crt}$<br>$t \in \{1, \dots, T\}$<br>$u_{cr} \overset{\text{i.i.d.}}{\sim} N(0, \sigma_u^2), \epsilon_{crt} \overset{\text{i.i.d.}}{\sim} N(0, \sigma^2)$
2	**Null hypothesis**	*Reduced model:*<br>$f(t) = \beta_0 + \sum_b s_b(t_i)$<br>$0 \leq t \leq T$	*Reduced model:*<br>$f(t) = \frac{1 - p}{1 + e^{-\left(\frac{a}{t} - b\right)}} + p$<br>$0 \leq t \leq T$	$DIM = \sum_{t=0}^{T} f_1(t) - \sum_{t=0}^{T} f_0(t) = 0$<br>$0 \leq t \leq T$	$DIM = \sum_{t \in \{6,7,8\}} \mu_{1t} - \sum_{t \in \{6,7,8\}} \mu_{0t} = 0$
3	**Moderation**	mandatory	mandatory	none	optional
4	**Post-processing filters**	none	Keep if:<br>1. R2 > 0.8<br>2. sigmoid plateau <0.3	none	none
5	**Model-based inference**	$F = \frac{\frac{RSS_0 - RSS_1}{d_1}}{\frac{RSS_1}{\tilde{s}_i}} \overset{H_0}{\sim} F(d_1, d_0 + d_2)$	$F = \frac{\frac{RSS_0 - RSS_1}{\sigma_0^2}}{\frac{RSS_1}{\sigma_0^2}} \overset{H_0}{\sim} F(d_1, d_2)$	$z = \frac{\widehat{DIM}}{se(\widehat{DIM})} \sim N(0,1)$	$t = \frac{\widehat{DIM}}{se(\widehat{DIM})} \sim Student_d$

Table 2. **Statistical modeling.** Statistical modeling and inference are performed separately for each protein, therefore we omit protein index subscript p for simplicity. In Supplemental Table 2 Step 1, TPP and Shape-Constrained Additive Models (SCAM) rely on splines, while NPARC fits a sigmoid curve. TPP and NPARC do not distinguish the variation between biological replicates and residual error. SCAM incorporate a monotonicity constraint and distinguish the variation between biological replicates and residual error. MSstatsTMT discards the reference channel used for normalization (t=0), and fits a non-parametric curve where each temperature has its own expected value. It distinguishes the variation between biological replicates and residual error. In Supplemental Table 2 step 2, TPP and NPARC specify null hypotheses in terms of reduced (i.e., simplified) models, while SCAM and MSstatsTMT specify the null hypotheses in terms of differences in estimated means (DIM) for all (SCAM) or a subset of (MSstatsTMT) temperatures. In Supplemental Table 2, Steps 3, TPP and NPARC require a moderation step to characterize the non-systematic variation. In Supplemental Table 2, Step 4, NPARC implements additional post-processing filtering. In Supplemental Table 2, Step 5, hypotheses are tested using summaries of the data (i.e., test statistics), compared to reference probability distributions.

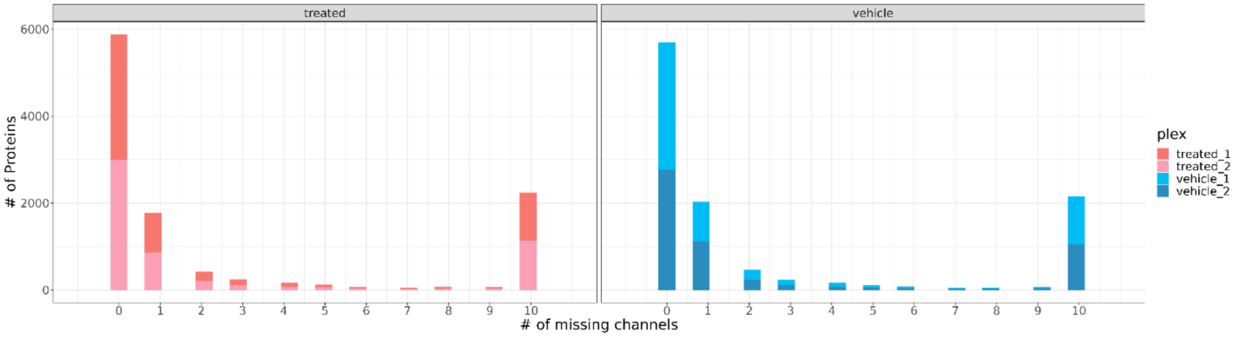

Figure S-1. **Dataset 1- Phaneuf *et al.*, histogram of proteins listed with missing channels per plex (vignette 1, section A).** X-axis: number of missing channels in a plex. Y-axis: the number of unique protein groups with that number of missing channels in a plex in the PD protein-level summary. Colors: plexes.

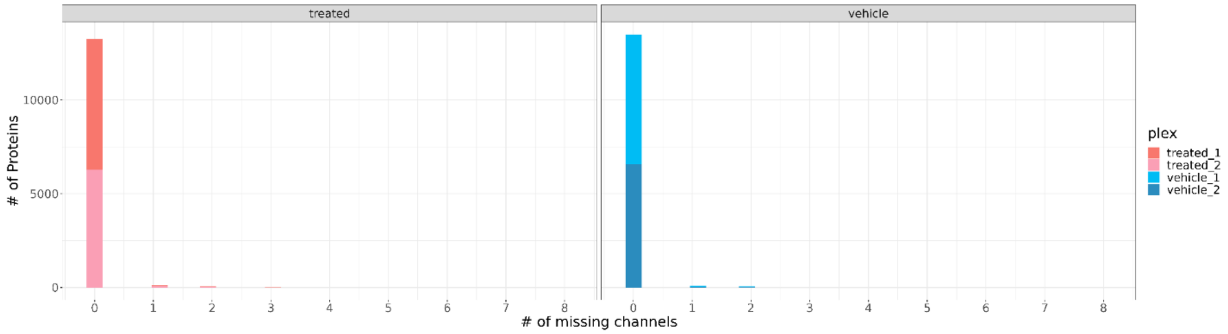

Figure S-2. **Dataset 2- Leijten *et al.*, histogram of proteins listed with missing channels per plex (vignette 3, section A)**. X-axis: number of missing channels in a plex in the PD protein-level summary. Y-axis: the number of proteins with that number of missing channels in a plex. Colors: plexes.

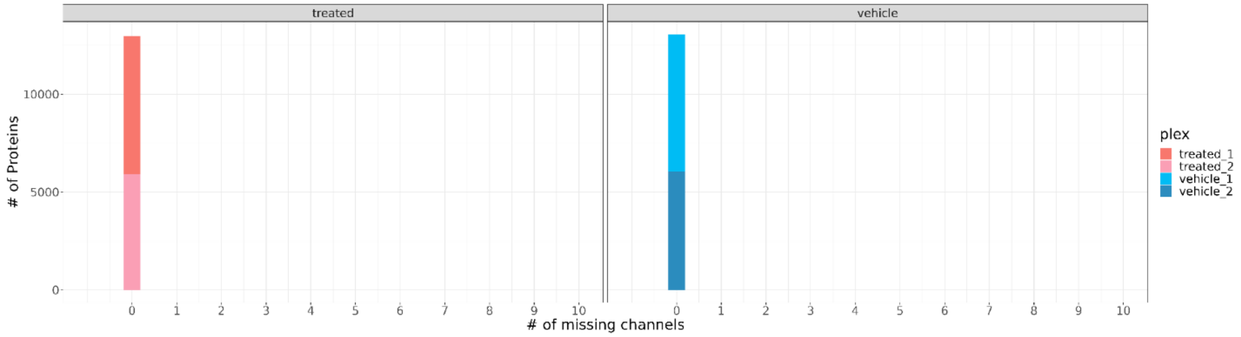

Figure S-3. **Dataset 3a- Xu *et al.*, histogram of proteins listed with missing channels per plex (vignette 5, section A).** X-axis: number of missing channels in a plex in the PD protein-level summary. Y-axis: the number of proteins with that number of missing channels in a plex. Colors: plexes.

Algorithm 1: Simulate log-feature intensities in a thermal profile

Input:

T // Number of temperatures. In this manuscript T=10

C // Number of conditions. In this manuscript C=2, 0 is vehicle, treated is 1.

R // Number of biological replicates per condition. In this manuscript R=2.

$\hat{f}_c(t)$ // $\hat{f}_c(t)$ is a T-dimensional vector representing population-level protein step function on the log scale. In this manuscript, the template is a protein selected from the experimental data, normalized, and fit with MSstatsTMT. For an interacting protein, $\hat{f}_1(t) \neq \hat{f}_2(t)$. For a non-interacting protein, $\hat{f}_1(t) = \hat{f}_2(t)$.

σ_{tech}= median($\sigma_{tech}{}^{dataset\ 1}$)// Residual (within subject) standard deviation.

ICC // intra-class correlation coefficient. In this manuscript, ICC={5%, 40%)

Output:

Y_{crt}^{obs}, c= {0,1} r= {1,R} t={1,...,T}//Output is simulated on the log-scale.

Algorithm:

$\sigma_{bio}^2 = \frac{\sigma_{tech}^2}{\frac{1}{ICC}-1}$ // biological variation corresponding to the desired ICC

For each c = 0,1

For each r = 1...R

Sample $u_{cr} \sim N(0, \sigma_{bio}^2)$ // between-subjects variation

For each t = 1 ... T

$Y_{crt}^{true} = \hat{f}_c(t)$

Sample $\epsilon_{crt} \sim N(0, \sigma_{tech}^2 = 0.267)$ // within-subject variation

$Y_{crt}^{obs} = Y_{crt}^{true} + u_{cr} + \epsilon_{crt}$

Return: Y_{crt}^{obs}

Figure S-4. **Pseudocode for simulating thermal proteome profiling datasets with varying percentages of biological variability**

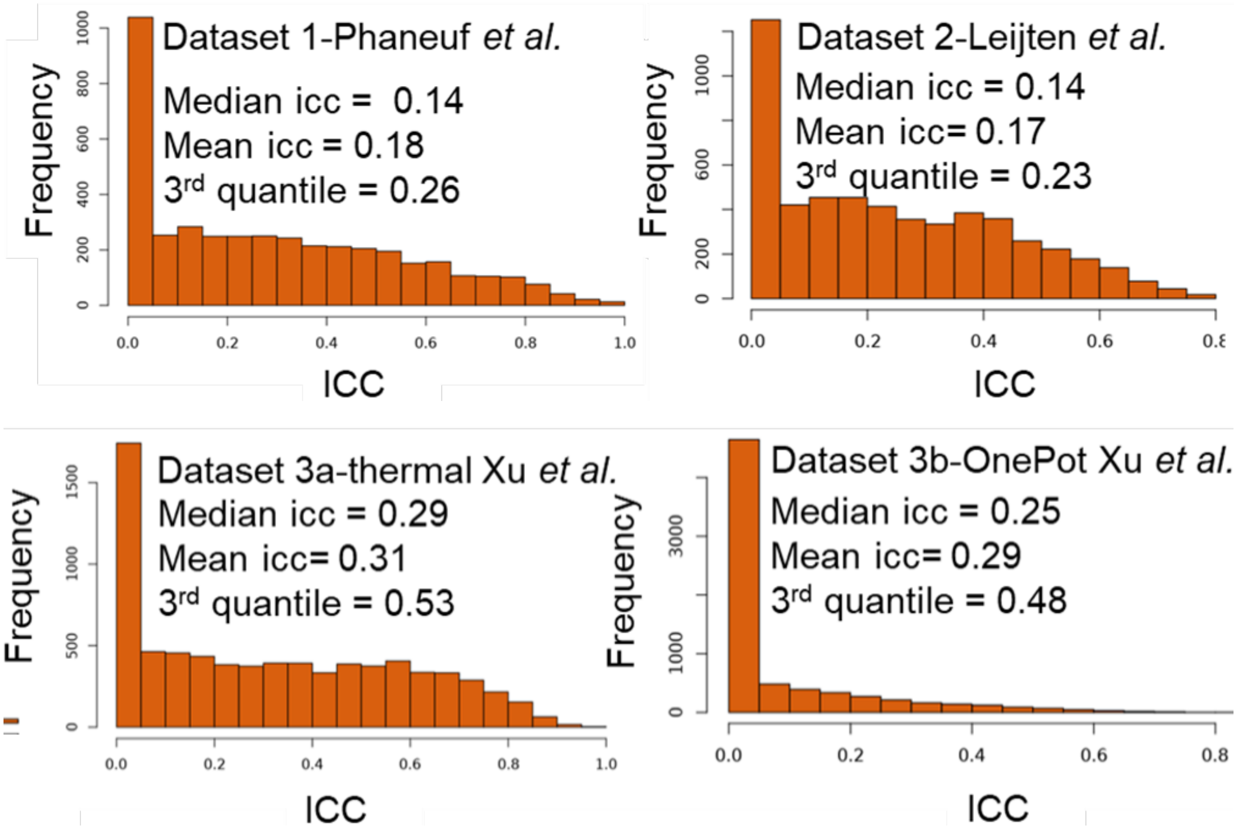

Figure S-5. Interclass correlation coefficient (ICC) distributions in the experimental datasets(vignette 10). ICC of a protein is defined as the proportion of biological between-replicate variation in the total variation (Equation 1 in the main manuscript). It was estimated by the MSstatsTMT model fit after the MSstatsTMT processing (Supplemental Table 1, column 2).

Algorithm 2: Simulate log-feature intensities in a thermal pool

```
Input:
        Y_crt^obs // Full curve thermal profiling output from Algorithm 1
Output:
Y_pcr^onePot c= {0,1} r= {1,...,R} //Output is simulated on the log-scale
Algorithm:
For each c = 0,1
        For each r = 1...R
                X_crt^obs = 2^(Y_crt^obs) // unlog values
                X_cr^onePot = X̄_cr.^obs // average abundances across temperatures
                Y_cr^onePot = log_2(X_cr^onePot) // log the resulted summaries
Return: Y_cr^onePot
```

Figure S-6. **Pseudocode for simulating thermal proteome profiling pool datasets (vignette 11)**

2. Background

2.1 Data structures and notation

A

Protein	Spectrum File (Run/Condition/Mixture)	File ID (r)	Abundance 126	Abundance 127N	Abundance 127C	Abundance 128N	Abundance 128C	Abundance 129N	Abundance 129C	Abundance 130N	Abundance 130C	Abundance 131
1	Vehicle_1.raw	F1	Y_{010}^{PD}	Y_{011}^{PD}	Y_{012}^{PD}	Y_{013}^{PD}	Y_{014}^{PD}	Y_{015}^{PD}	Y_{016}^{PD}	Y_{017}^{PD}	Y_{018}^{PD}	Y_{019}^{PD}
1	Vehicle_2.raw	F2	Y_{020}^{PD}	Y_{021}^{PD}	Y_{022}^{PD}	Y_{023}^{PD}	Y_{024}^{PD}	Y_{025}^{PD}	Y_{026}^{PD}	Y_{027}^{PD}	Y_{028}^{PD}	Y_{029}^{PD}
1	Treated_1.raw	F3	Y_{110}^{PD}	Y_{111}^{PD}	Y_{112}^{PD}	Y_{113}^{PD}	Y_{114}^{PD}	Y_{115}^{PD}	Y_{116}^{PD}	Y_{117}^{PD}	Y_{118}^{PD}	Y_{119}^{PD}
1	Treated_2.raw	F4	Y_{120}^{PD}	Y_{121}^{PD}	Y_{122}^{PD}	Y_{123}^{PD}	Y_{124}^{PD}	Y_{125}^{PD}	Y_{126}^{PD}	Y_{127}^{PD}	Y_{128}^{PD}	Y_{129}^{PD}

B

Protein	Spectrum File (Run/Condition/Mixture)	File ID (r)	Abundance 126	Abundance 127N	Abundance 127C	Abundance 128N	Abundance 128C	Abundance 129N	Abundance 129C	Abundance 130N	Abundance 130C	Abundance 131
1	Vehicle_1.raw	F1	Y_{0101}^{PD}	Y_{0111}^{PD}	Y_{0121}^{PD}	Y_{0131}^{PD}	Y_{0141}^{PD}	Y_{0151}^{PD}	Y_{0161}^{PD}	Y_{0171}^{PD}	Y_{0181}^{PD}	Y_{0191}^{PD}
1	Vehicle_1.raw	F1	Y_{0102}^{PD}	Y_{0112}^{PD}	Y_{0122}^{PD}	Y_{0132}^{PD}	Y_{0142}^{PD}	Y_{0152}^{PD}	Y_{0162}^{PD}	Y_{0172}^{PD}	Y_{0182}^{PD}	Y_{0192}^{PD}
1	Vehicle_2.raw	F2	Y_{0201}^{PD}	Y_{0211}^{PD}	Y_{0221}^{PD}	Y_{0231}^{PD}	Y_{0241}^{PD}	Y_{0251}^{PD}	Y_{0261}^{PD}	Y_{0271}^{PD}	Y_{0281}^{PD}	Y_{0291}^{PD}
1	Vehicle_2.raw	F2	Y_{0202}^{PD}	Y_{0212}^{PD}	Y_{0222}^{PD}	Y_{0232}^{PD}	Y_{0242}^{PD}	Y_{0252}^{PD}	Y_{0262}^{PD}	Y_{0272}^{PD}	Y_{0282}^{PD}	Y_{0292}^{PD}
1	Treated_1.raw	F3	Y_{1101}^{PD}	Y_{1111}^{PD}	Y_{1121}^{PD}	Y_{1131}^{PD}	Y_{1141}^{PD}	Y_{1151}^{PD}	Y_{1161}^{PD}	Y_{1171}^{PD}	Y_{1181}^{PD}	Y_{1191}^{PD}
1	Treated_1.raw	F3	Y_{1102}^{PD}	Y_{1112}^{PD}	Y_{1122}^{PD}	Y_{1132}^{PD}	Y_{1142}^{PD}	Y_{1152}^{PD}	Y_{1162}^{PD}	Y_{1172}^{PD}	Y_{1182}^{PD}	Y_{1192}^{PD}
1	Treated_2.raw	F4	Y_{1201}^{PD}	Y_{1211}^{PD}	Y_{1221}^{PD}	Y_{1231}^{PD}	Y_{1241}^{PD}	Y_{1251}^{PD}	Y_{1261}^{PD}	Y_{1271}^{PD}	Y_{1281}^{PD}	Y_{1291}^{PD}
1	Treated_2.raw	F4	Y_{1202}^{PD}	Y_{1212}^{PD}	Y_{1222}^{PD}	Y_{1232}^{PD}	Y_{1242}^{PD}	Y_{1252}^{PD}	Y_{1262}^{PD}	Y_{1272}^{PD}	Y_{1282}^{PD}	Y_{1292}^{PD}

Table S-1. **General design of thermal proteome profiling experiments in this manuscript, for one protein.** Protein subscript p is omitted for simplicity. Rows are TMT plexes. Each TMT plex represents a biological replicate. The first two replicates are from the vehicle condition, and the last two replicates are from the treated condition. Columns are channels, assigned to distinct temperature treatments. Numbers in each cell are feature or protein abundances. Subscripts indicate condition c=1,2, replicate $r=1,\ldots,R$, temperatures $t=0,\ldots,T$ (t = 0 is the reference channel or lowest temperature) in the special case of R=4 and T=9. (A) Output of data processing at the protein level, where protein abundances are indicated by Y_{crt}^{PD}. *(B)* Output of data processing at the feature level, where feature intensities are indicated by X_{crti}^{PD}, $i=1,\ldots,I$ in the special case of I=2. Boxes indicate features of the same protein within each plex. Rows within each box indicate distinct features from the same protein.

2.2 Data processing methods for thermal proteome profiling

Algorithm 3: Normalization factor calculation algorithm

Input:

Y'_{pcrt} // Protein-level abundances, produced by a data processing tool such as Proteome Discoverer, ratioed to the lowest temperature

// $p = \{1,\ldots,P\}$, $c = \{0,1\}$ $r = \{1,\ldots,R\}$ $t = \{0,\ldots,T\}$, $Y''_{pcr0} = 1$ (t=0 is the reference channel or the lowest temperature)

Output:

Q_t // Normalization factor for each t

Algorithm:

//NormSet keeps proteins where ratios at t=6, 8 and 9 have no outliers in any condition and any replicate

NormSet = $\{ p \mid (Y'_{pcr6}) \in [0.6, 0.9] \cap (Y'_{pcr8}) \leq 0.3 \cap (Y'_{pcr9}) \leq 0.2 \; for\ all\ c\ and\ all\ r \}$

For each c = 0,1

For each r =1,…,R

For each t=1,…,T

$Y''_{crt} = \underset{p \in NormSet}{\text{median}} (Y'_{pcrt})$ /// For each c, r and t, calculate the median over all proteins in NormSet

$\hat{Y}''_{crt} = \frac{1-\hat{m}}{1+e^{-\left(\frac{\hat{a}}{t}-\hat{b}\right)}} + \hat{m}$ // For each c and r, fit a sigmoid curve to the median ratioed abundances

$R^2_{cr} = 1 - \frac{\sum_{t=1}^{T}(Y''_{crt}-\hat{Y}''_{crt})^2}{\sum_{t=1}^{T}(Y''_{crt}-\bar{Y}''_{crt})^2}$ // For each c and r, calculate the R^2 of the sigmoid fit to the median ratioed abundances

$c^*, r^* = \arg\max_{c,r} R^2_{cr}$ // Select condition and replicate for which the fitted sigmoid curve maximizes R^2

$Q_t = \left(\frac{\hat{Y}''_{c^*r^*t}}{Y''_{c^*r^*t}}\right)$ // Compute normalization vector

Return: (Q_t)

Figure S-7. **Pseudocode describing TPP processing normalization steps.**

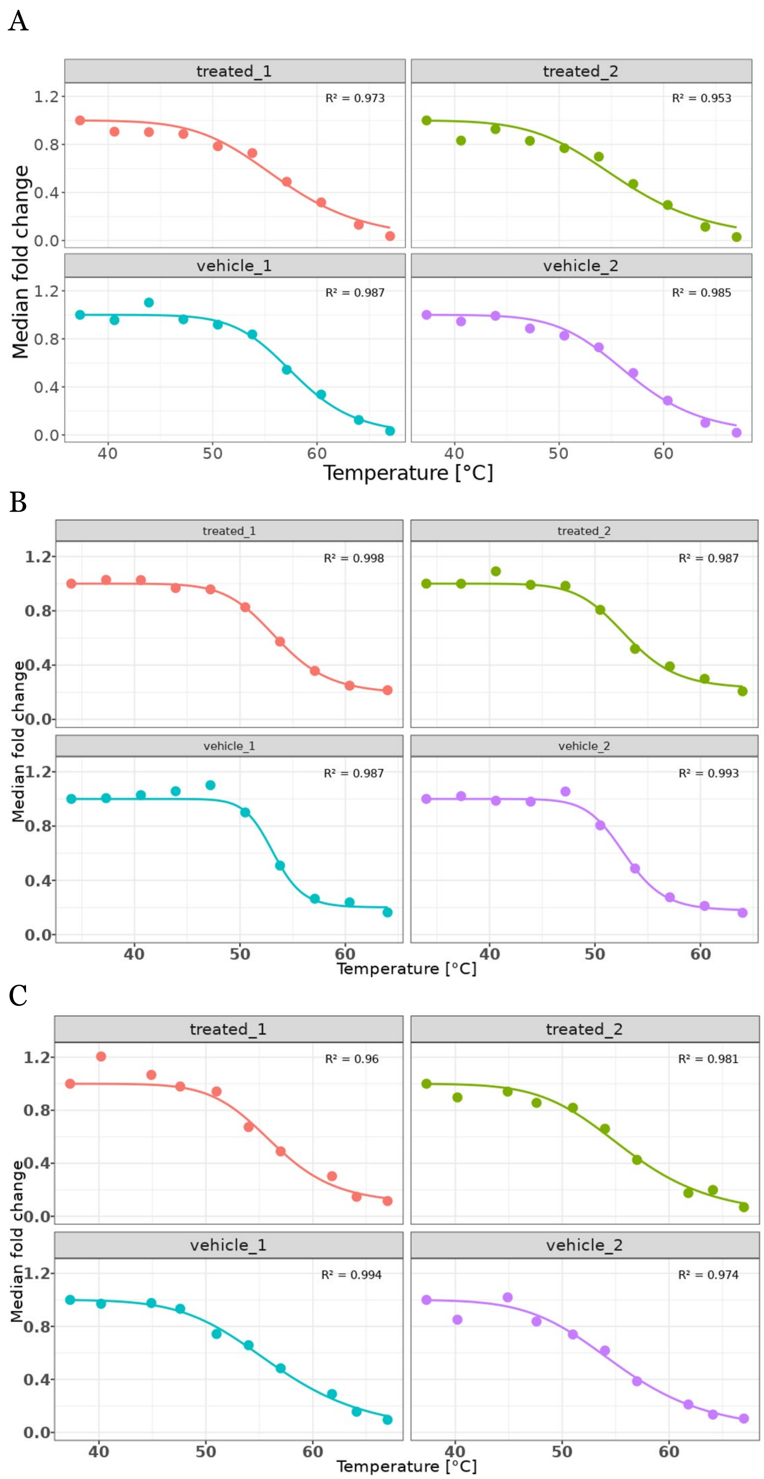

Figure S-8. **Normalization curves fitted by TPP, separately for each biological replicate**. Dots are median values from all the proteins in NormSet calculated at each temperature (line 12 in Supplemental Figure S-7). Lines are sigmoid curves fitted to the median values (line 13 in Supplemental Figure S-7). (A): Dataset 1, 378 proteins NormSet. (B): Dataset 2, 183 proteins in NormSet. (C) Dataset 3, 215 proteins in NormSet.

2.3 Statistical models for thermal proteome profiling

Column		1	2	3	4
**Step**	**Statistical model**	**TPP (version 3.26.0)**	**NPARC (version 1.10.1)**	**SCAM splines (version 1.2-13)**	**MSstatsTMT (version 2.6.0)**
**1**	**Model**	$Y_{crt} = f_c(t) + \epsilon_{crt}$<br>$f_c(t) = \frac{1 - p_{cr}}{1 + e^{-\left(\frac{a_{cr}}{t} - b_{cr}\right)}} + p_{cr}$<br>$0 \leq t \leq T$<br>$\epsilon_{crt} \overset{\text{i.i.d.}}{\sim} N(0, \sigma^2)$	$Y_{crt} = f_c(t) + \epsilon_{crt}$<br>$f_c(t) = \frac{1 - p_c}{1 + e^{-\left(\frac{a_c}{t} - b_c\right)}} + p_c$<br>$0 \leq t \leq T$<br>$\epsilon_{crt} \overset{\text{i.i.d.}}{\sim} N(0, \sigma^2)$	$Y_{crt} = f_c(t) + u_{cr} + \epsilon_{crt}$<br>$f_c(t) = \beta_{0c} + \sum_b s_{bc}(t), \frac{df_c(t)}{dt} \leq 0$<br>$0 \leq t \leq T$<br>$u_{cr} \overset{\text{i.i.d.}}{\sim} N(0, \sigma_u^2), \epsilon_{crt} \overset{\text{i.i.d.}}{\sim} N(0, \sigma^2)$	$Y_{crt} = \mu_{ct} + u_{cr} + \epsilon_{crt}$<br>$t \in \{1, \ldots, T\}$<br>$u_{cr} \overset{\text{i.i.d.}}{\sim} N(0, \sigma_u^2), \epsilon_{crt} \overset{\text{i.i.d.}}{\sim} N(0, \sigma^2)$
**2**	**Null hypothesis**	*Reduced model:*<br>$T_{m,cr} = \frac{1 - a_{cr}}{b - \ln(\frac{1 - p_{cr}}{0.5 - p_{cr}} - 1)} + b_{cr}$<br>$H_0: \Delta T_{m,r} = T_{m,1r} - T_{m,0r} = 0$<br>$0 \leq t \leq T$	*Reduced model:*<br>$f\ (t) = \frac{1 - p}{1 + e^{-\left(\frac{a}{t} - b\right)}} + p$<br>$0 \leq t \leq T$	$DIM = \sum_{t=0}^{T} f_1(t) - \sum_{t=0}^{T} f_0(t) = 0$<br>$0 \leq t \leq T$	$DIM = \sum_{t \in \{6,7,8\}} \mu_{1t} - \sum_{t \in \{6,7,8\}} \mu_{0t} = 0$
**3**	**Moderation**	mandatory	mandatory	none	optional
**4**	**Post-processing filters**	Keep if:<br>1. R2 > 0.8<br>2. sigmoid plateau <0.3	Keep if:<br>1. R2 > 0.8<br>2. sigmoid plateau <0.3	none	none
**5**	**Model-based inference**	Subjective criteria of a high-quality fit based on quality of fit and empirically derived p -values	$F = \frac{\frac{RSS_0 - RSS_1}{\sigma_0^2}}{\frac{RSS_1}{\sigma_0^2}} \overset{H_0}{\sim} F(d_1, d_2)$	$z = \frac{\widehat{DIM}}{se(\widehat{DIM})} \sim N(0,1)$	$t = \frac{\widehat{DIM}}{se(\widehat{DIM})} \sim Student_d$

Table S-2. Statistical modeling. The table repeats Supplemental Table 2 from, while describing the sigmoid-based approach in TPP. Statistical modeling and inference are performed separately for each protein, therefore we omit subscript p for simplicity. TPP and NPARC fit a sigmoid curve, while Shape-Constrained Additive Models (SCAM) rely on splines. TPP and NPARC and TPP do not distinguish the variation between biological replicates and residual error. SCAM incorporate a monotonicity constraint and distinguish the variation between biological replicates and residual error. MSstatsTMT discards the reference channel used for normalization (t=0), and fits a non-parametric curve where each temperature has its own expected value. Both SCAM and MSstatsTMT specify the test statistic in terms of difference in means (DIM) for all (SCAM) or a subset of (MSstatsTMT) temperatures.

TPP sigmoid model assumptions Supplemental Table S-2 repeats Supplemental Table 2 from the main manuscript, while summarizing the TPP approach based on a sigmoid curve fit. Parameters of the sigmoid (lower plateau p), and constants a and b) are fit separately for each condition and replicate.

TPP null hypothesis for the sigmoid fit The T_m value, i.e. the temperature at which half of the initial soluble protein abundance is recorded, is derived from each curve, separately for each condition and replicate, as shown in

Supplemental Table S-2, line 2. The null hypothesis in Supplemental Table 2, Step 2 states that the difference between treated and vehicle $T_{m,cr}$ values (i.e. $\Delta T_{m,r}$) for each replicate is zero.

TPP model-based inference for the sigmoid fit $\Delta T_{m,r}$ for each replicate are converted into robust *z*-scores, with empirically determined means and standard deviations. The *z*-scores are compared to the Standard Normal distribution to determine p-values. There have been multiple attempts across TPP versions to determine criteria of a high quality fit, and criteria that distinguish protein interactions[3-4]. Earlier implementations suggested to keep proteins where the minimum *p*-values between replicates were less than 0.1 and maximum *p*-values were less than 0.2, the $\Delta T_{m,r}$ values had the same sign between replicates, the $\Delta T_{m,r}$ values between treated and vehicle conditions were greater than the $\Delta T_{m,r}$ values between vehicle replicates, and the minimum slope values at the T_{mr} were less than – 0.06[5]. Most of these filters have been removed from recent versions of the TPP package, and the guidance on *p*-value-based filtering is not well defined[6]. Filtering based on R^2 and lower sigmoid plateau were suggested by the original publication, and thus were implemented in this manuscript as shown in Supplemental Table S-2, Step 4 and Step 5.[5]

Supplemental Figures S-9(A) and Supplemental Figures S-10(A) illustrate the TPP sigmoid fit in the special case of proteins MAP2K1 and MAP2K2 from Dataset 1.

3. Evaluation: Detection of protein interactors was impacted by both data processing method and choice of statistical model

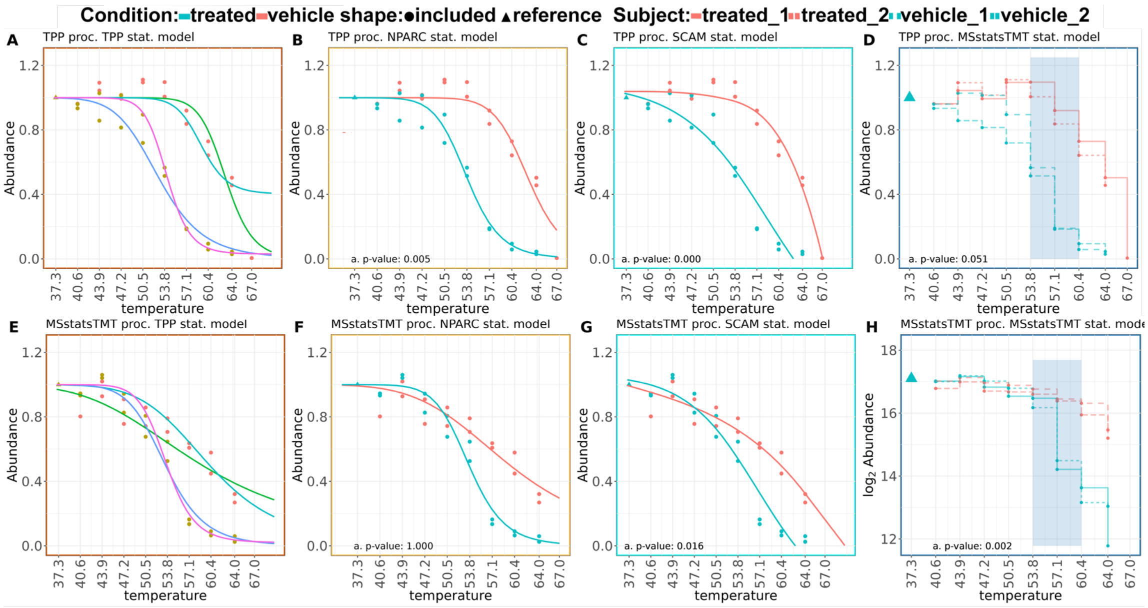

Figure S-9. **Dataset 1- Phaneuf *et al.*, known interactor MAP2K1 with TPP 3.26 (sigmoid fit, vignette 1 section M and vignette 2 section N).** A-D: TPP processing as in Supplemental Table 1, column 1.E-H: MSstatsTMT processing as in Supplemental Table 1, column 2. A and E: TPP statistical model as in Table S-2 column 1, B and F: NPARC statistical model as in Table S-2 column 2,C and G: SCAM model as in Table S-2 column 3, D and H: MSstatsTMT statistical model as in Table S-2, column 4.Different processing and modeling strategies produce different curves and different hypothesis testing results.

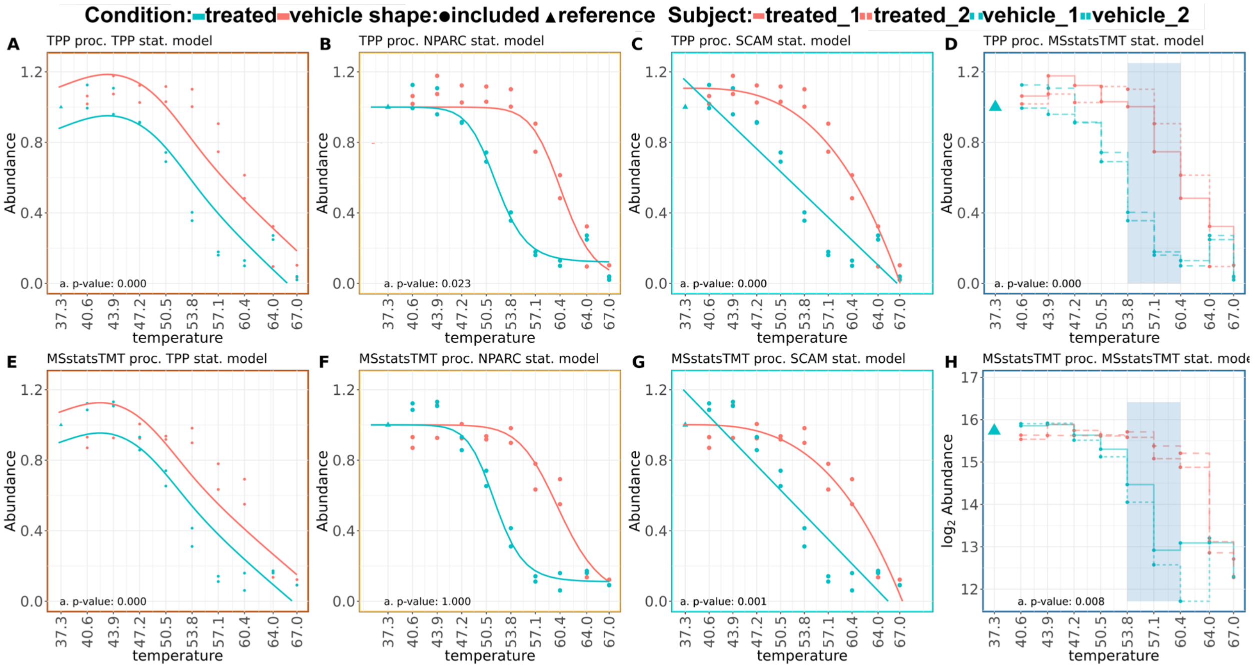

Figure S-10. **Dataset 1- Phaneuf *et al.*, observed data and fitted curves for known interactor MAP2K2 (vignettes 1 and 2, section J).** A-D: TPP processing (splines fit as in Supplemental Table 1, column 1). E-H: MSstatsTMT processing (splines fit as in Supplemental Table 1, column 2). A and E: TPP statistical model (spline fit as in Supplemental Table 2, column 1), B and F: NPARC statistical model as in Supplemental Table 2, column 2, C and G: SCAM model as in Supplemental Table 2, column 3. D and H: MSstatsTMT statistical model as in Supplemental Table 2, column 4. Line types emphasize the distinct biological replicates per each condition. Triangles indicate reference channels used for normalization by MSstatsTMT. Blue areas indicate subsets of temperatures used by the null hypothesis (Step 2 in Supplemental Table 2, column 4).

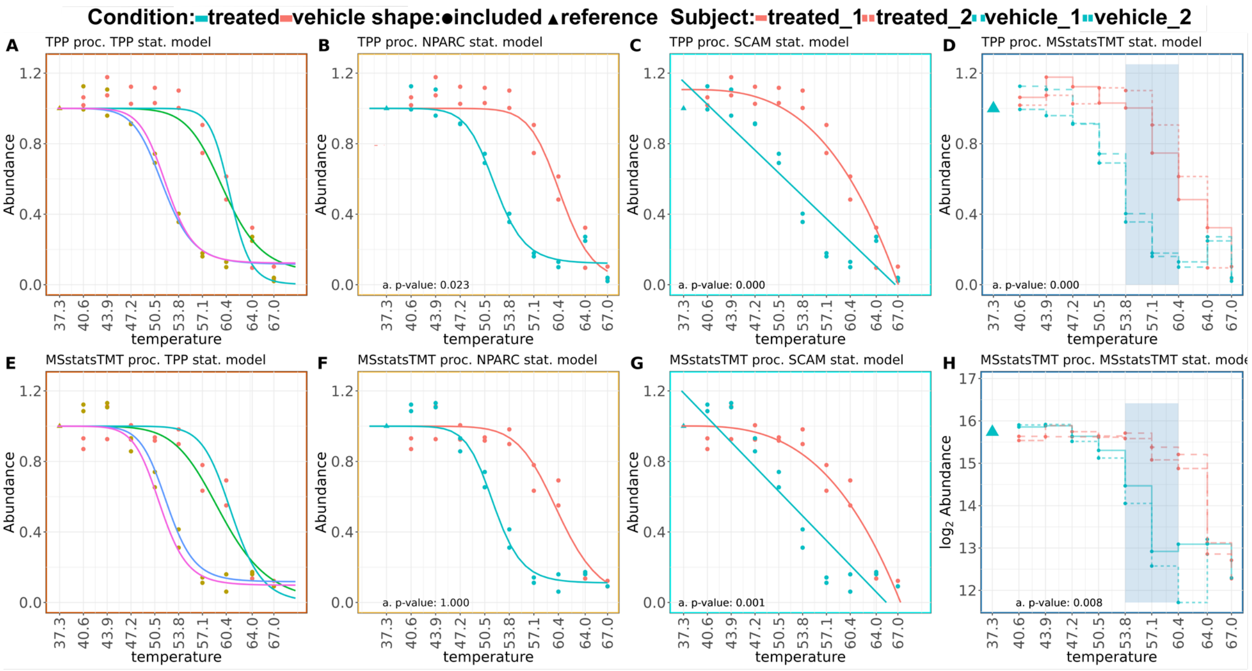

Figure S-11. **Dataset 1- Phaneuf *et al.*, observed data and fitted curves for known interactor MAP2K2.** Same as shown in Figure S-10, vignette 1 section M and vignette 2, section N, except A and E: TPP statistical model (sigmoid fit, separately for each replicate as in Table S-2, column 1).

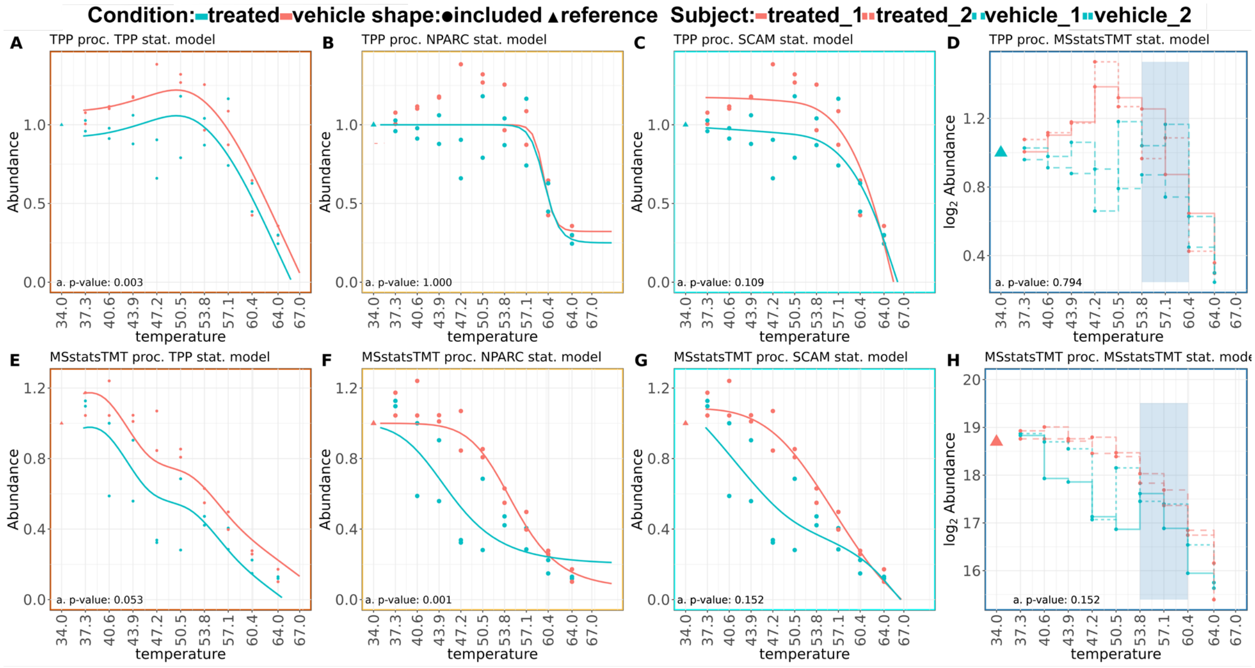

Figure S-12. **Dataset 2- Leijten *et al.*, observed data and fitted curves for known interactor ALDH1A3 (vignette 3, section I and vignette 4, section K).** A-D: TPP processing (splines fit as in Supplemental Table 1, column 1). E-H: MSstatsTMT processing (splines fit as in Supplemental Table 1, column 2). A and E: TPP statistical model (spline fit as in Supplemental Table 2, column 1), B and F: NPARC statistical model as in Supplemental Table 2, column 2, C and G: SCAM model as in Supplemental Table 2, column 3. D and H: MSstatsTMT statistical model as in Supplemental Table 2, column 4. Line types emphasize the distinct biological replicates per each condition. Triangles indicate reference channels used for normalization by MSstatsTMT. Blue areas indicate subsets of temperatures used by the null hypothesis (Step 2 in Supplemental Table 2, column 4).

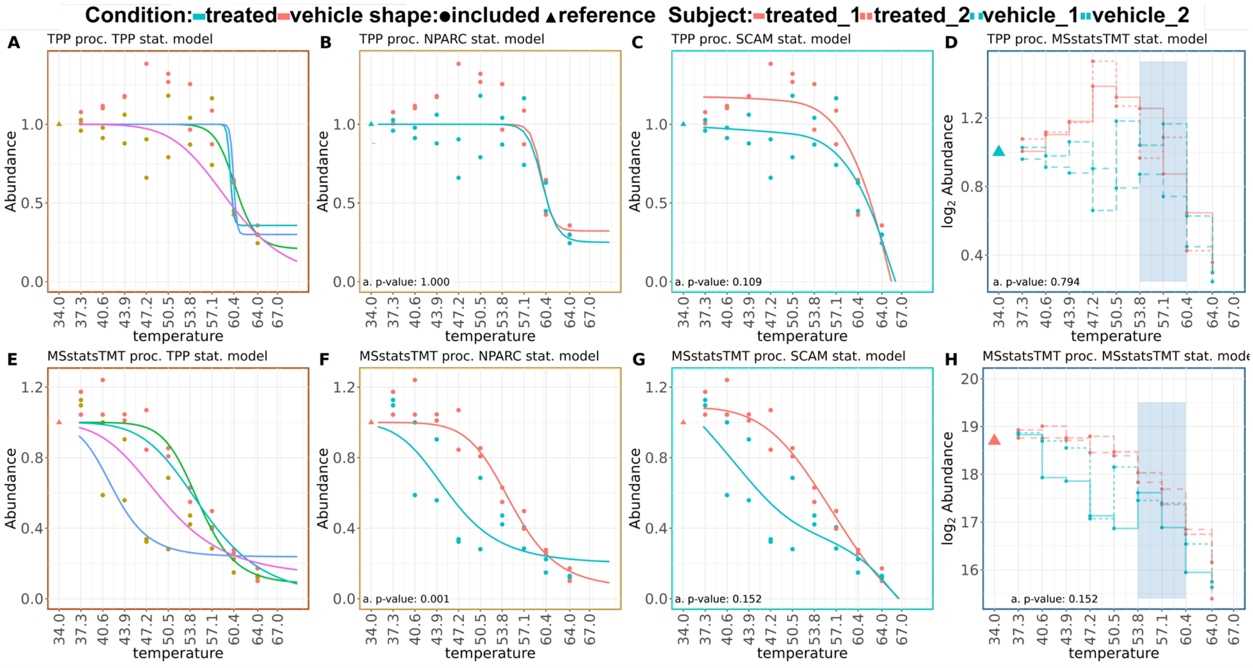

Figure S-13. **Dataset 2- Leijten *et al.*, protein interactor ALDH1A3 processing affects statistical conclusion for NPARC (vignettes 3 section N and vignette 4, section P).** Same as in Fig. S-12 except A and E: sigmoid fit, separately for each replicate as in Table S-2, column 1. A-D: TPP processing. E-H: MSstatsTMT processing. A and E: TPP statistical model (sigmoid fit), B and F: NPARC statistical model, C and G: SCAM model, D and H: MSstatsTMT statistical model. Different processing and modeling strategies produce different curves and different hypothesis testing results for NPARC and SCAM.

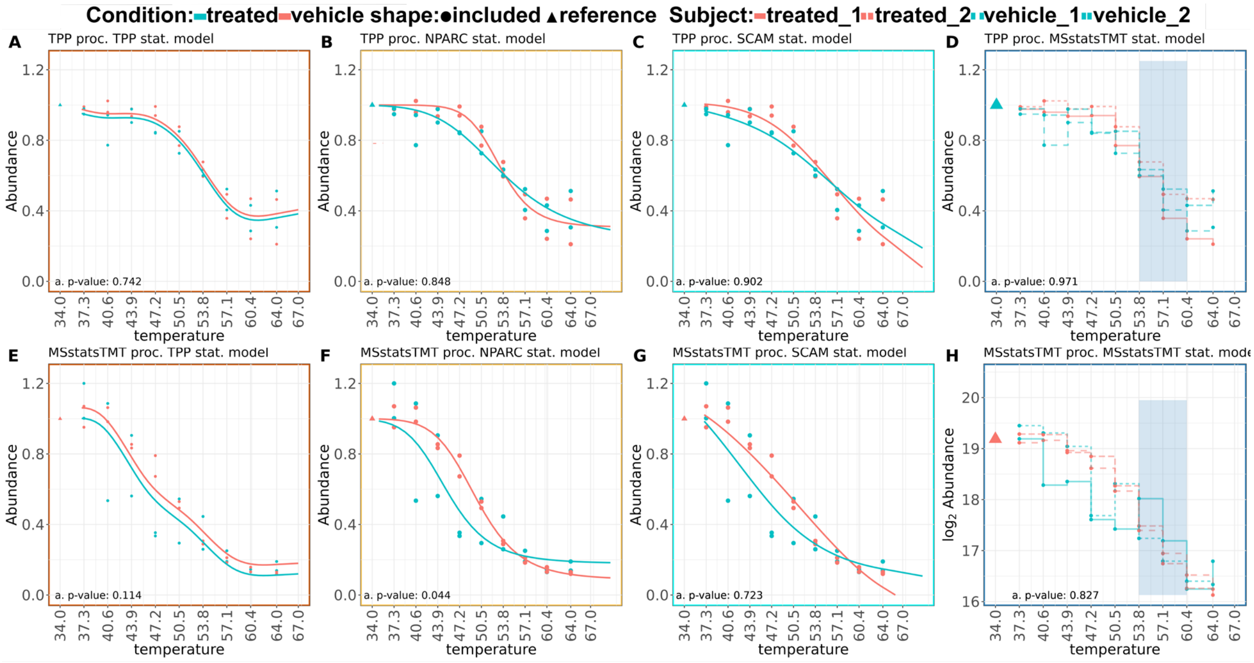

Figure S-14. **Dataset 2- Leijten *et al.*, non-interactor STAT3 difference in processing affects profiles and NPARC statistical conclusions (vignette 3, section I and vignette 4, section K).** A-D: TPP processing (splines fit as in Supplemental Table 1, column 1). E-H: MSstatsTMT processing (splines fit as in Supplemental Table 1, column 2). A and E: TPP statistical model (spline fit as in Supplemental Table 2, column 1), B and F: NPARC statistical model as in Supplemental Table 2, column 2, C and G: SCAM model as in Supplemental Table 2, column 3. D and H: MSstatsTMT statistical model as in Supplemental Table 2, column 4. Line types emphasize the distinct biological replicates per each condition. Triangles indicate reference channels used for normalization by MSstatsTMT. Blue areas indicate subsets of temperatures used by the null hypothesis (Step 2 in Supplemental Table 2, column 4). Only MSstatsTMT processing and NPARC statistical model show interaction.

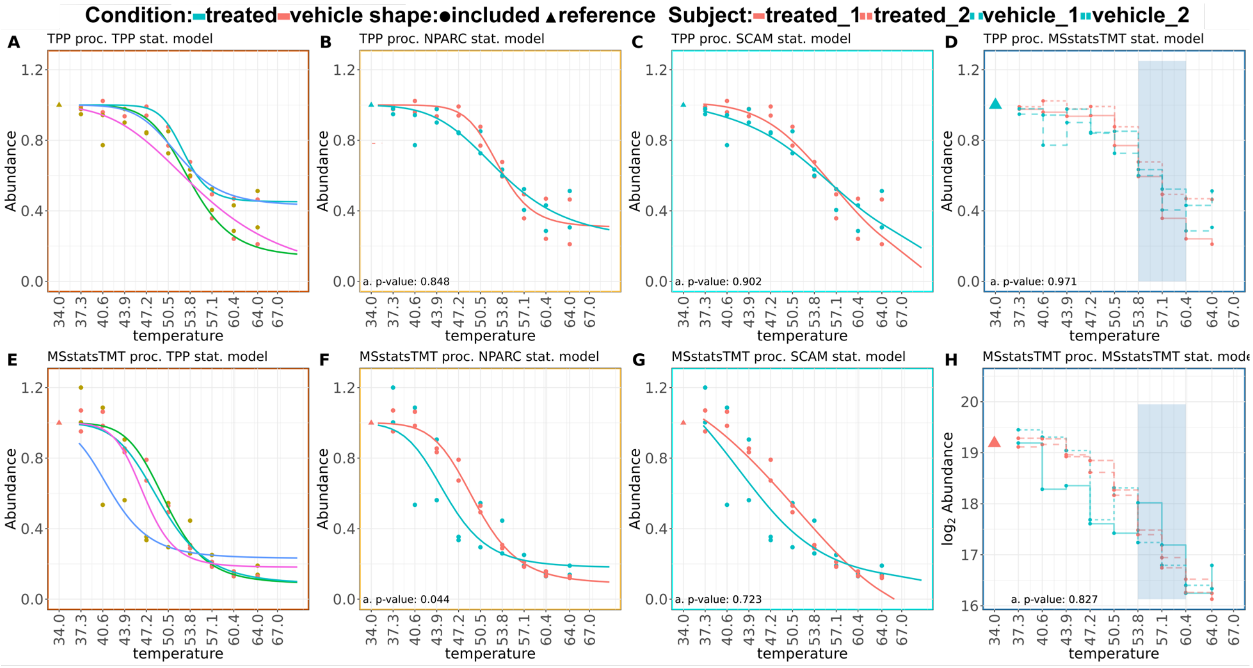

Figure S-15. **Dataset 2- Leijten *et al.*, non-interactor STAT3 where processing affects profiles and statistical conclusions for NPARC (vignette 3 section N and vignette 4 section P).** Same as in Fig. S-14 except A and E: sigmoid fit, separately for each replicate as in Table S-2, column 1. A-D: TPP processing. E-H: MSstatsTMT processing. A and E: TPP statistical model (sigmoid fit), B and F: NPARC statistical model, C and G: SCAM model, D and H: MSstatsTMT statistical model. Different processing and modeling strategies produce different curves and different hypothesis testing results.

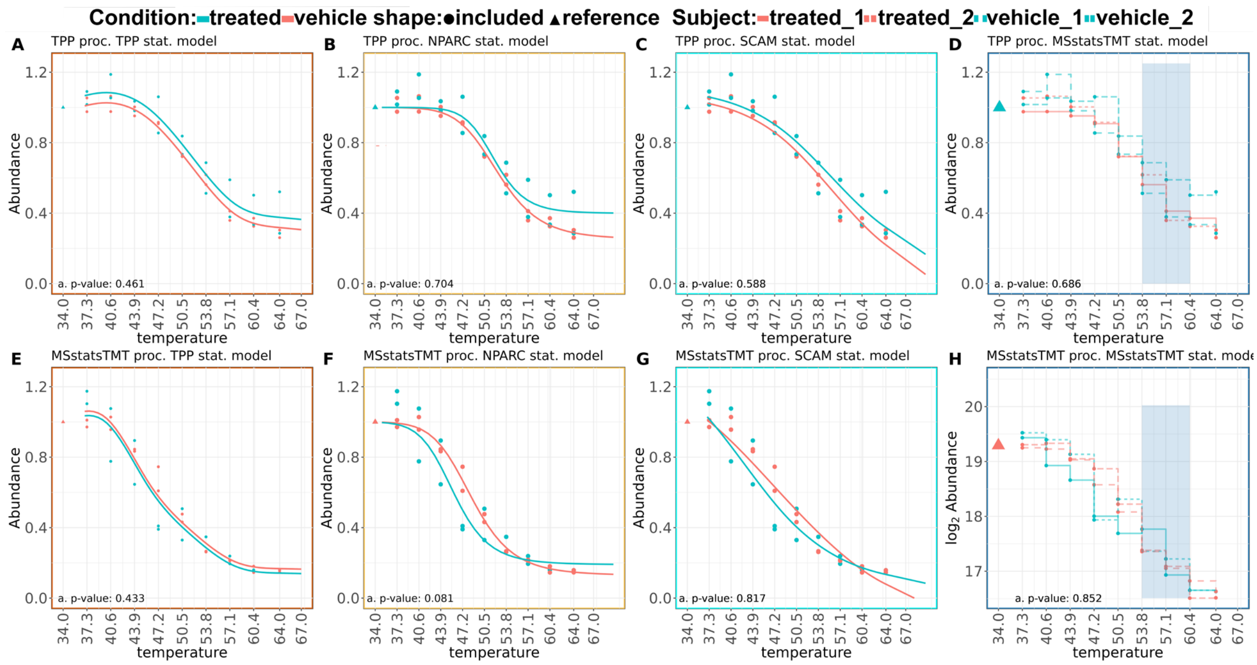

Figure S-16. **Dataset 2- Leijten *et al.*, non-interactor STAT5 where processing affects profiles and statistical conclusions for NPARC (vignette 3, section I and vignette 4, section K).** A-D: TPP processing (splines fit as in Supplemental Table 1, column 1). E-H: MSstatsTMT processing (splines fit as in Supplemental Table 1, column 2). A and E: TPP statistical model (spline fit as in Supplemental Table 2, column 1), B and F: NPARC statistical model as in Supplemental Table 2, column 2, C and G: SCAM model as in Supplemental Table 2, column 3. D and H: MSstatsTMT statistical model as in Supplemental Table 2, column 4. Line types emphasize the distinct biological replicates per each condition. Triangles indicate reference channels used for normalization by MSstatsTMT. Blue areas indicate subsets of temperatures used by the null hypothesis (Step 2 in Supplemental Table 2, column 4). Different processing and modeling strategies produce different curves and different hypothesis testing results.

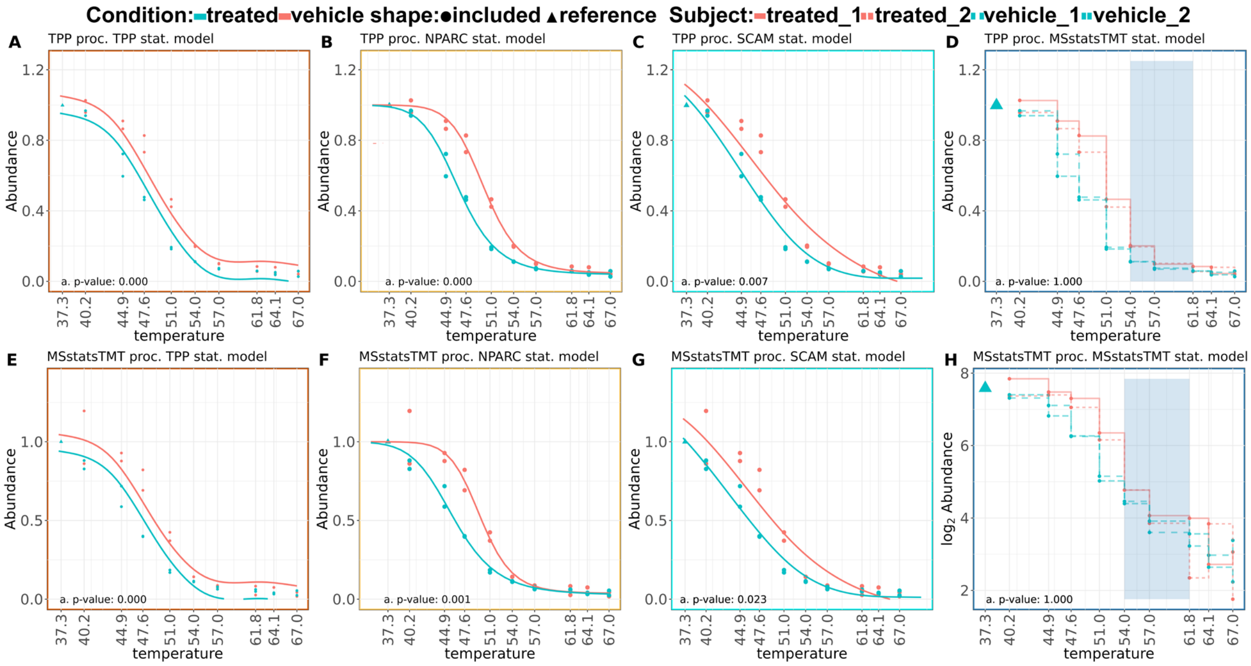

Figure S-17. **Dataset 3a- Xu *et al.*, interacting protein MAP2K2 with TPP 3.26 (spline fit, vignettes 5 and 6, section J).** A-D: TPP processing (splines fit as in Supplemental Table 1, column 1). E-H: MSstatsTMT processing (splines fit as in Supplemental Table 1, column 2). A and E: TPP statistical model (spline fit as in Supplemental Table 2, column 1), B and F: NPARC statistical model as in Supplemental Table 2, column 2, C and G: SCAM model as in Supplemental Table 2, column 3. D and H: MSstatsTMT statistical model as in Supplemental Table 2, column 4. Line types emphasize the distinct biological replicates per each condition. Triangles indicate reference channels used for normalization by MSstatsTMT. Blue areas indicate subsets of temperatures used by the null hypothesis (Step 2 in Supplemental Table 2, column 4). Different processing and modeling strategies produce different curves and different hypothesis testing results.

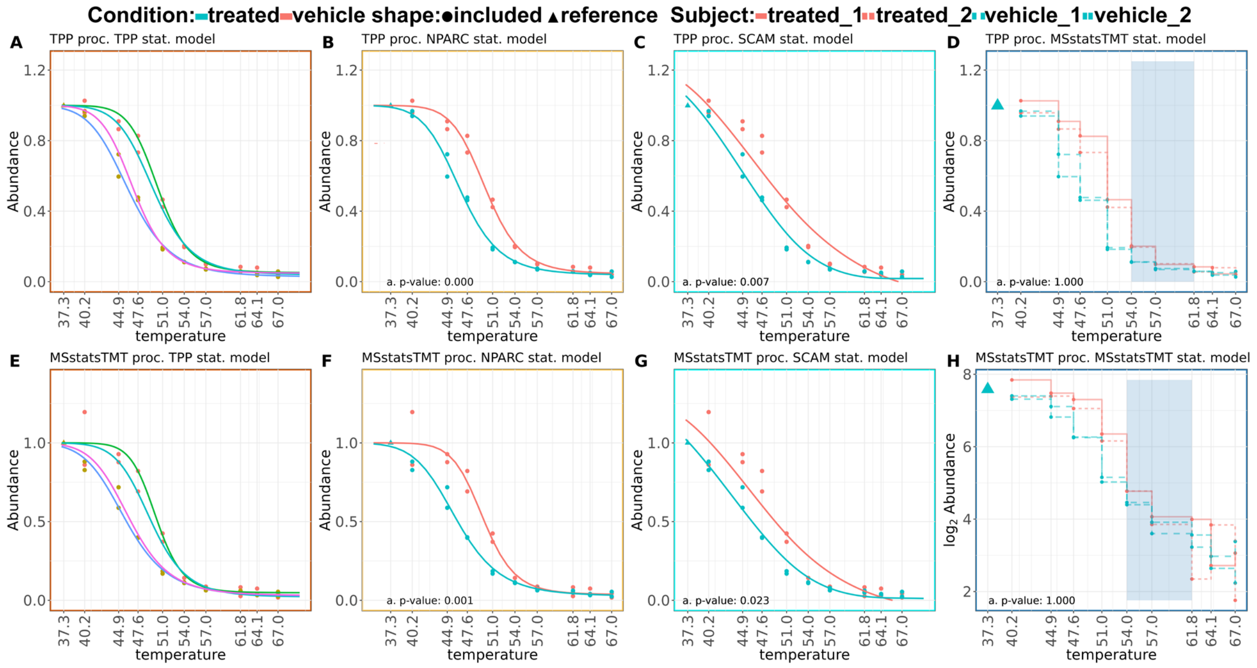

Figure S-18. **Dataset 3a- Xu *et al.*, interacting protein MAP2K2 with TPP 3.26 (sigmoid fit, vignettes 5 and 6, section P).** Same as in Fig. S-17 except A and E: sigmoid fit, separately for each replicate as in Table S-2, column 1. A-D: TPP processing as in Supplemental Table 1, column 1. E-H: MSstatsTMT processing as in Supplemental Table 1, column 2. B and F: NPARC statistical model, C and G: SCAM model, D and H: MSstatsTMT statistical model. Different processing and modeling strategies produce different curves and different hypothesis testing results.

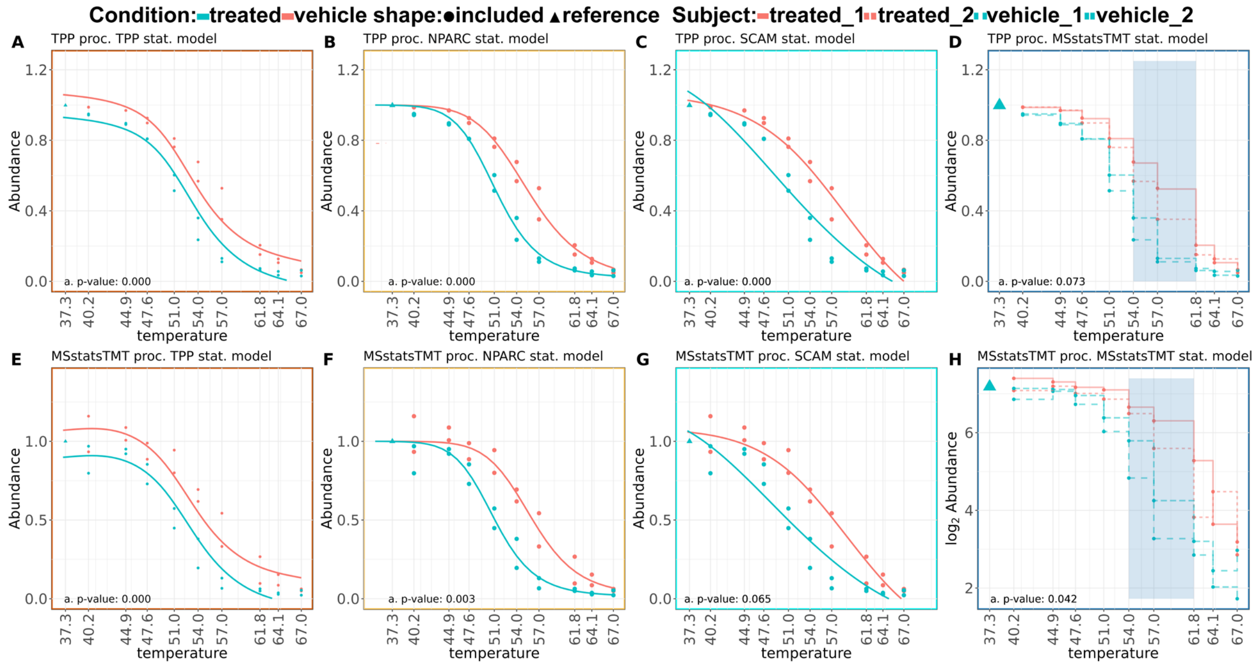

Figure S-19 **Dataset 3a- Xu *et al.*, interacting protein CHEK1 with TPP 3.26 (spline fit, vignettes 5 and 6, section J).** A-D: TPP processing (splines fit as in Supplemental Table 1, column 1). E-H: MSstatsTMT processing (splines fit as in Supplemental Table 1, column 2). A and E: TPP statistical model (spline fit as in Supplemental Table 2, column 1), B and F: NPARC statistical model as in Supplemental Table 2, column 2, C and G: SCAM model as in Supplemental Table 2, column 3. D and H: MSstatsTMT statistical model as in Supplemental Table 2, column 4. Line types emphasize the distinct biological replicates per each condition. Triangles indicate reference channels used for normalization by MSstatsTMT. Blue areas indicate subsets of temperatures used by the null hypothesis (Step 2 in Supplemental Table 2, column 4). Different processing and modeling strategies produce different curves and different hypothesis testing results.

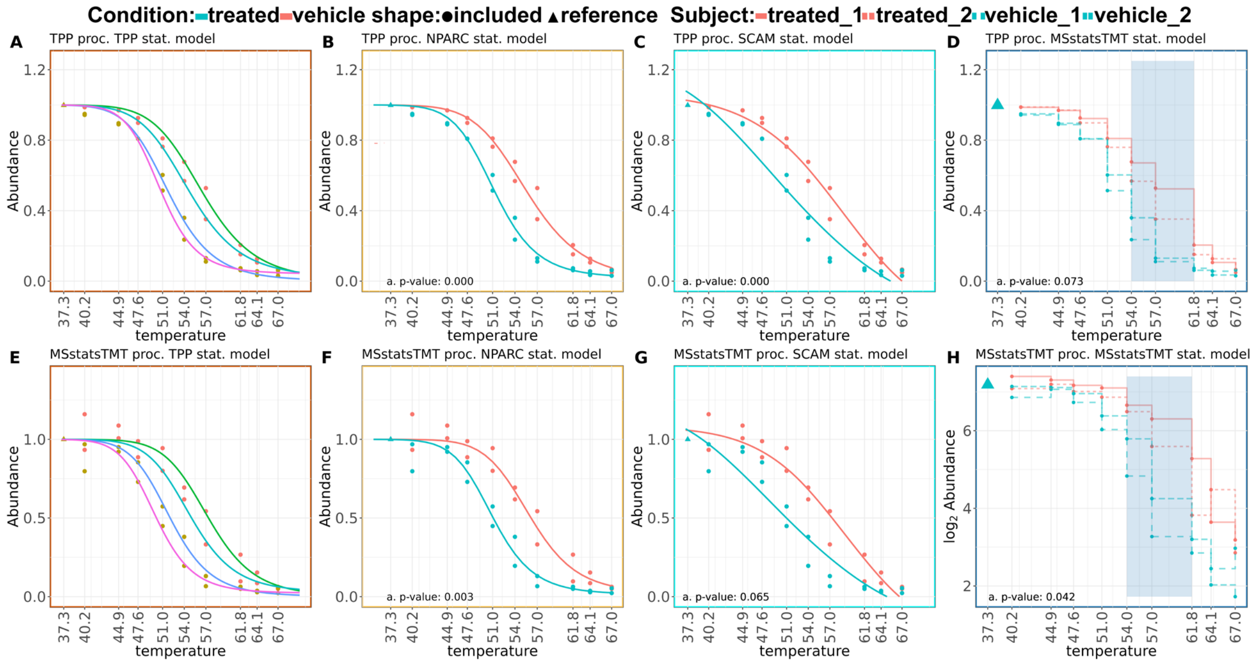

Figure S-20 **Dataset 3a- Xu *et al.*, interacting protein CHEK1 with TPP 3.26 (sigmoid fit, vignettes 5 and 6, section P).** Same as in Fig. S-19 except A and E: sigmoid fit, separately for each replicate as in Table S-2, column 1. A-D: TPP processing as in Supplemental Table 1, column 1. E-H: MSstatsTMT processing as in Supplemental Table 1, column 2. Different processing and modeling strategies produce different curves and different hypothesis testing results.

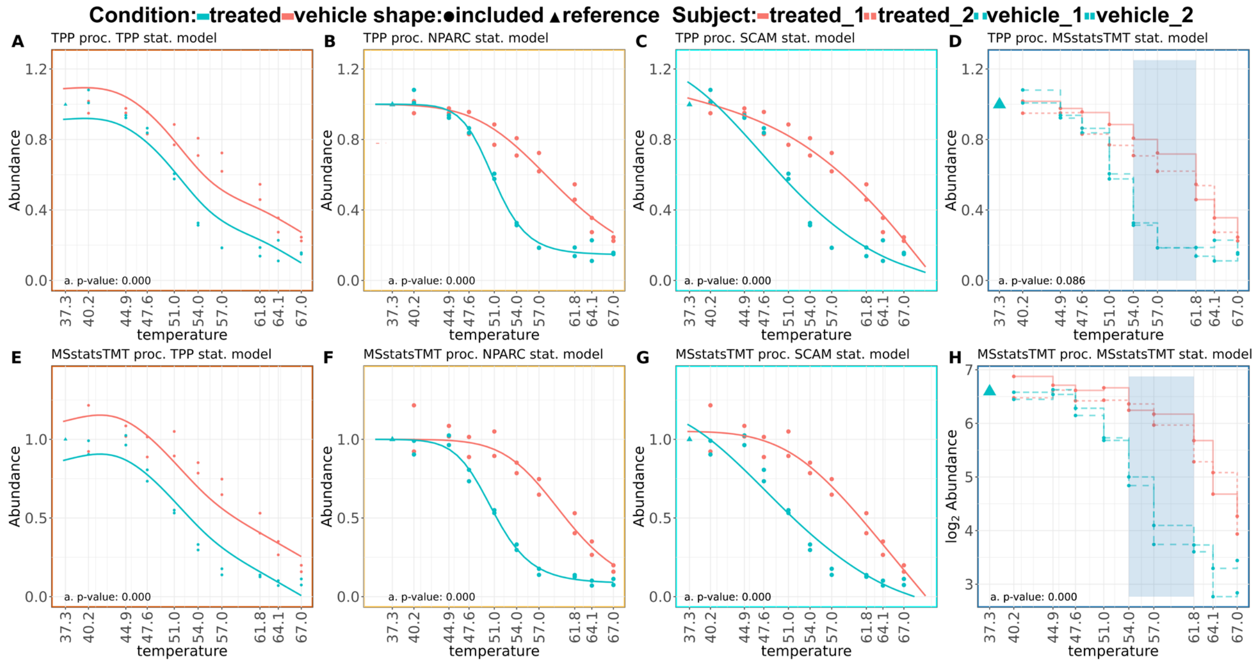

Figure S-21. **Dataset 3a- Xu *et al.*, interacting protein STK3 with TPP 3.26 (spline fit, vignettes 5 and 6, section J).** A-D: TPP processing (splines fit as in Supplemental Table 1, column 1). E-H: MSstatsTMT processing (splines fit as in Supplemental Table 1, column 2). A and E: TPP statistical model (spline fit as in Supplemental Table 2, column 1), B and F: NPARC statistical model as in Supplemental Table 2, column 2, C and G: SCAM model as in Supplemental Table 2, column 3. D and H: MSstatsTMT statistical model as in Supplemental Table 2, column 4. Line types emphasize the distinct biological replicates per each condition. Triangles indicate reference channels used for normalization by MSstatsTMT. Blue areas indicate subsets of temperatures used by the null hypothesis (Step 2 in Supplemental Table 2, column 4). Different processing and modeling strategies produce different curves and different hypothesis testing results.

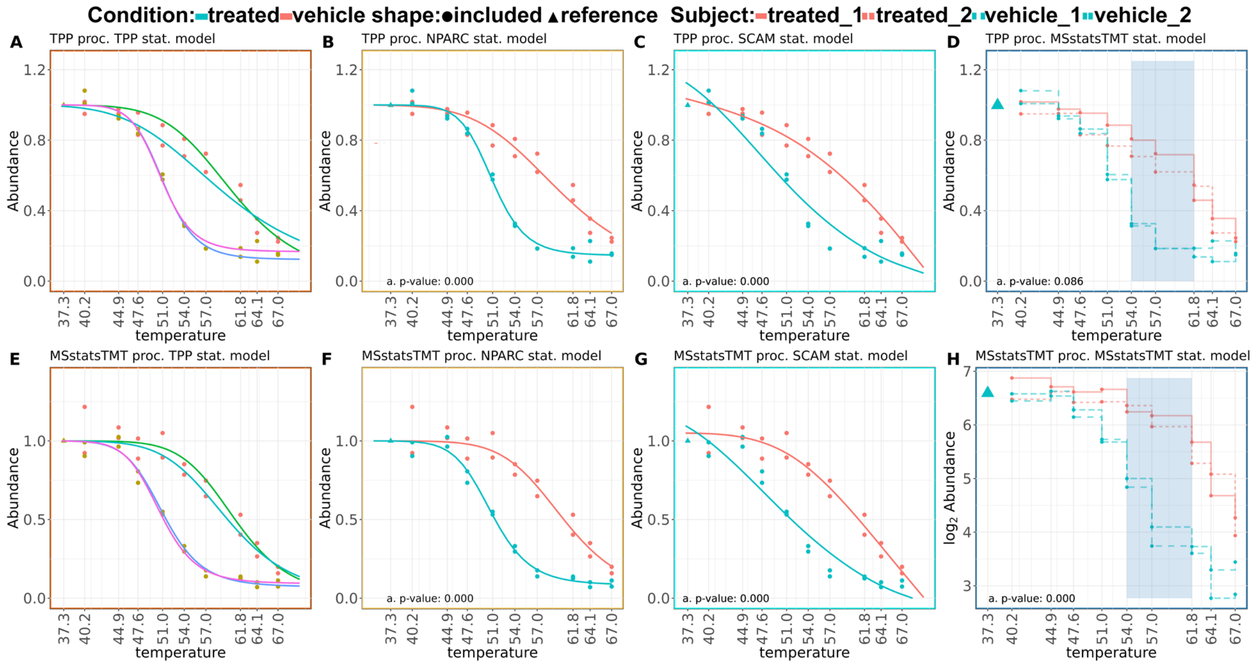

Figure S-22. **Dataset 3a- Xu *et al.*, interacting protein STK3 with TPP 3.26 (sigmoid fit, vignettes 5 and 6, section P).** Same as in Fig. S-17 except A and E: sigmoid fit, separately for each replicate as in Table S-2, column 1. A-D: TPP processing as in Supplemental Table 1, column 1. E-H: MSstatsTMT processing as in Supplemental Table 1, column 2. Different processing and modeling strategies produce different curves and different hypothesis testing results.

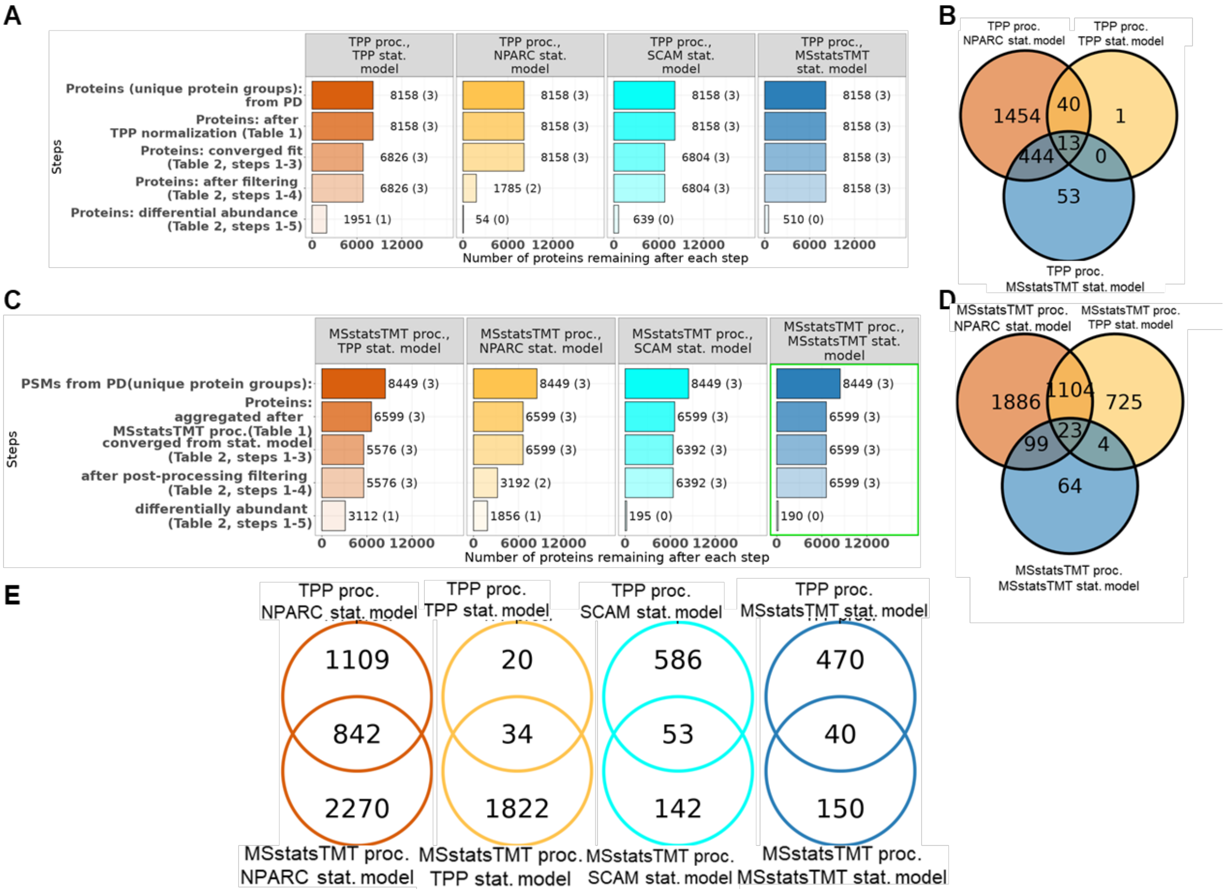

Figure S-23. **Dataset 2- Leijten *et al.* TPP protein interactions were not detected for all methods (vignette 3, sections J-L; and vignette 4, sections L-N).** A: TPP processing, steps are as in Supplemental Table 1, column 1. Each panel is a statistical model in Supplemental Table 2. Number of known protein interactions is in parentheses. B: Consistency of detecting protein interactions between statistical models, for a same data processing (TPP, spline fit). C: As in A, but with MSstatsTMT processing an in Supplemental Table 1, column 2. D: As in B, with MSstatsTMT processing. E: Consistency of detecting protein interactions between data processing strategies, for same statistical models. Decreased overlap between processing and statistical models as well as a failure to detect known protein interactors indicates different statistical conclusions.

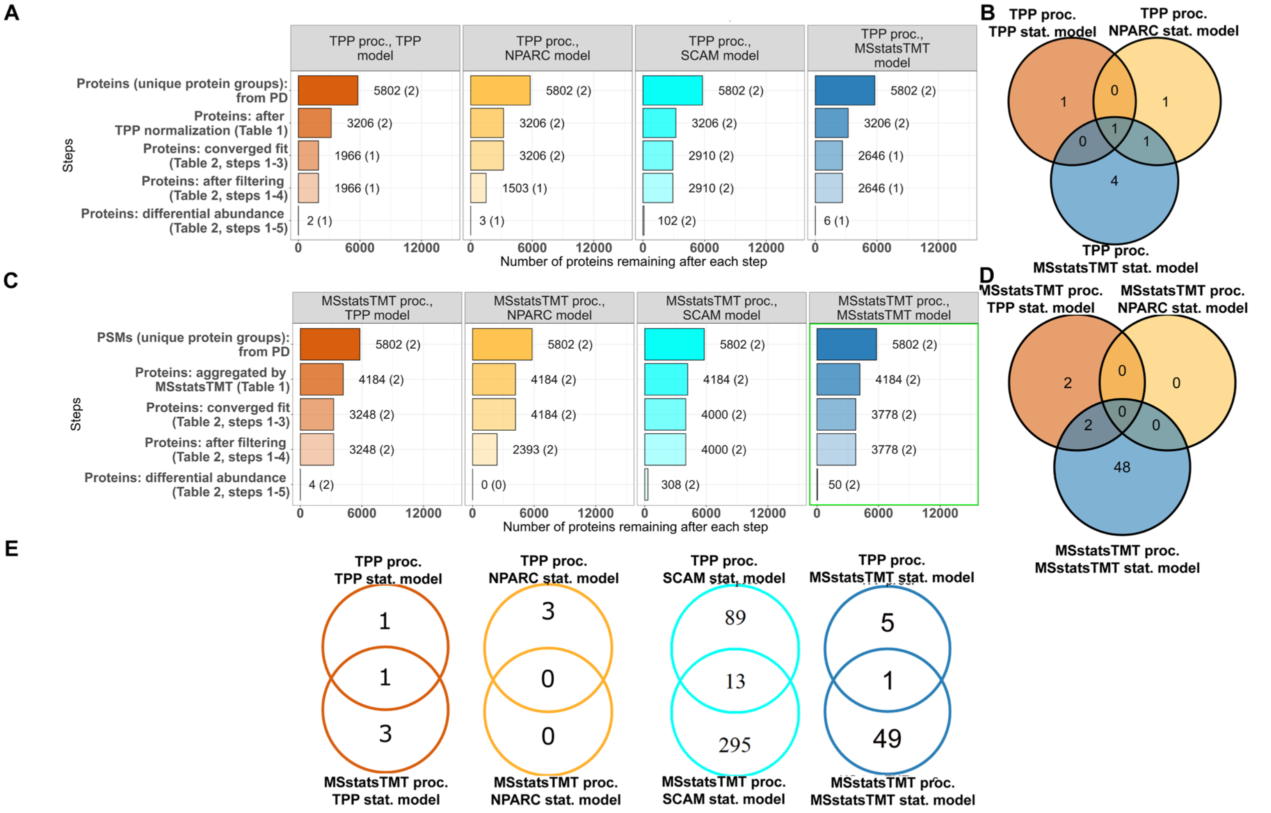

Figure S-24. **Dataset 1- Phaneuf *et al.*, processed with TPP 3.26 (sigmoid fit) detects less protein interactors (vignette 1, section L and 2, section M).** A: TPP processing, steps are as in Supplemental Table 1. Each column is a statistical model in Supplemental Table S-2. Number of known protein interactions is in parentheses. B: Consistency of detecting protein interactions between statistical models, for a same data processing (TPP). C: As in A, but with MSstatsTMT processing. D: As in B, with MSstatsTMT processing. E: Consistency of detecting protein interactions between data processing strategies, for same statistical models. MsstatsTMT loses sensitivity for known targets.

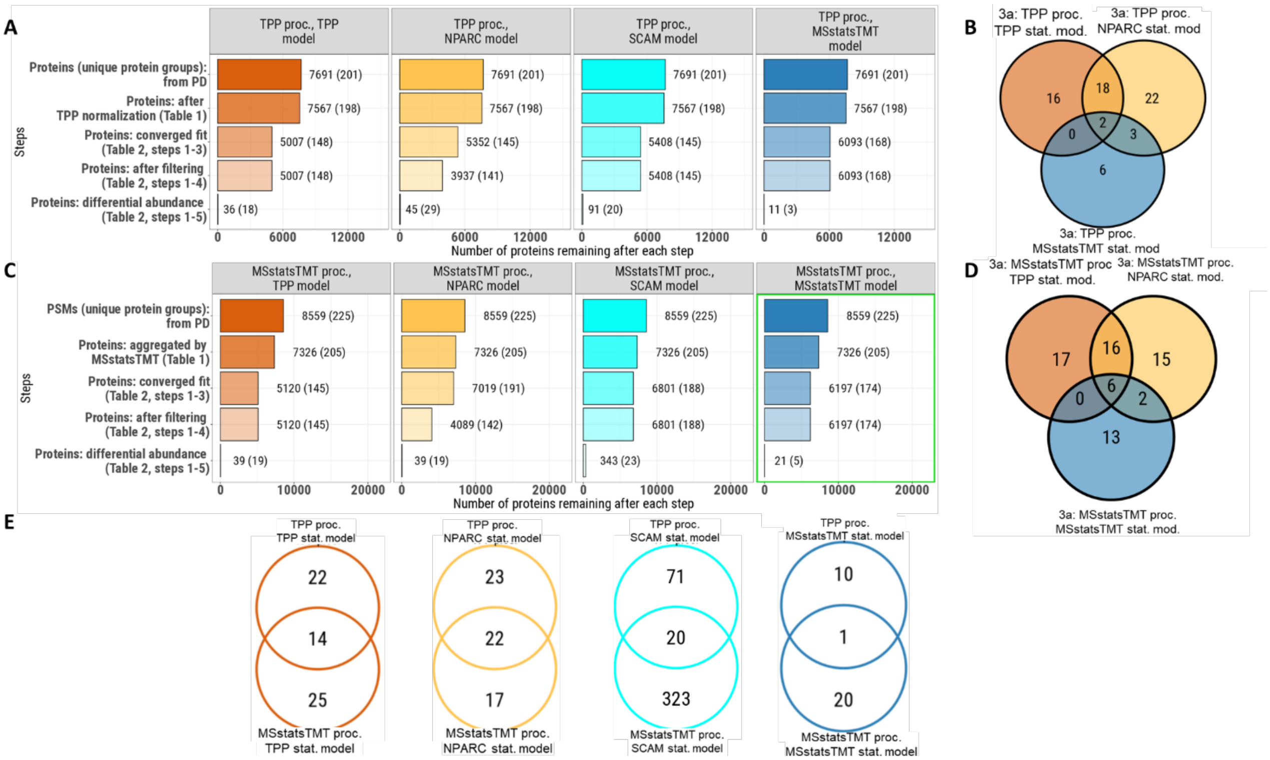

Figure S-25. **Dataset 3a- Xu *et al*, TPP version 3.26 (sigmoid fit) detects less kinases and interactors (sigmoid fit, vignettes 5 and 6, section Q).** A: TPP processing, steps are as in Supplemental Table S-7. Each column is a statistical model in Table S-2. Number of known protein interactions is in parentheses. B: Consistency of detecting protein interactions between statistical models, for a same data processing (TPP). C: As in A, but with MSstatsTMT processing. D: As in B, with MSstatsTMT processing. E: Consistency of detecting protein interactions between data processing strategies, for same statistical models. Lack of overlap between processing and statistical modeling are indicators of substantially different conclusions despite detecting some protein interactions.

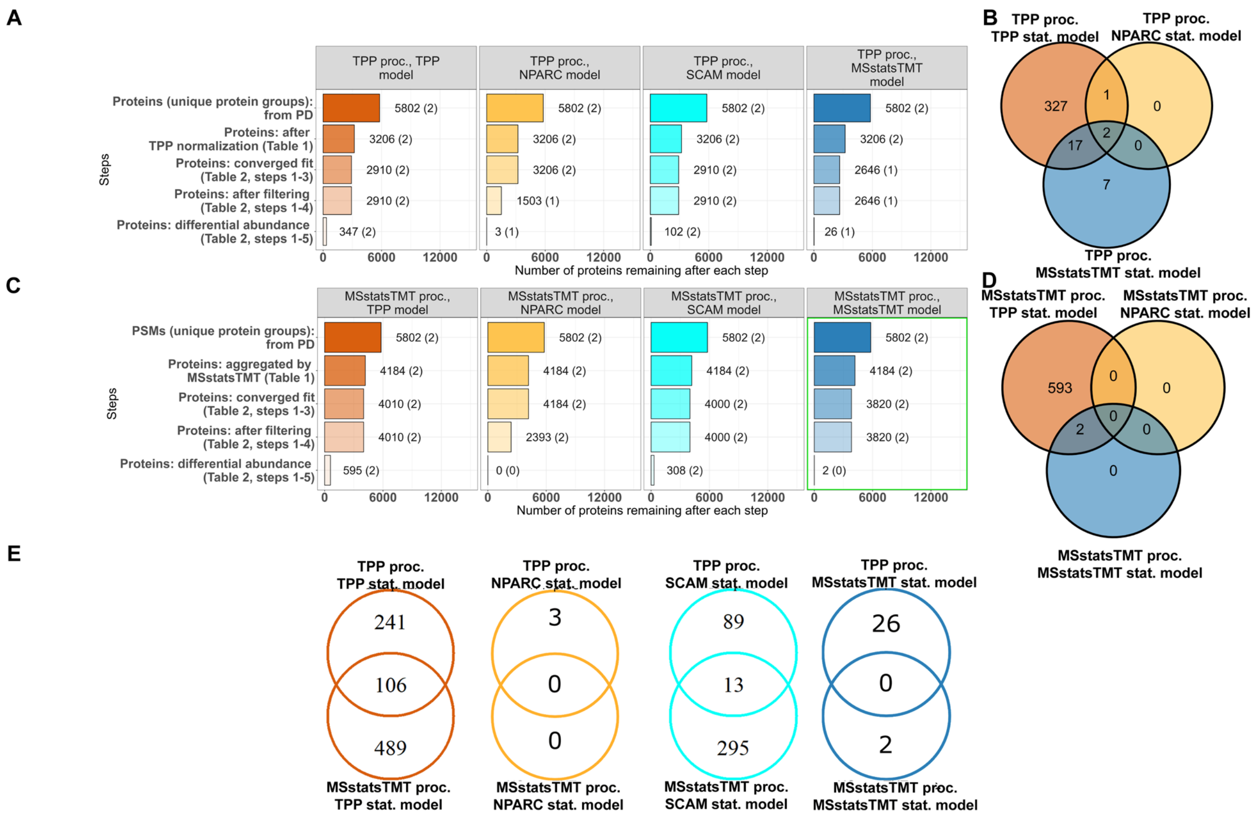

Figure S-26. **Dataset 1- Phaneuf *et al*, detected protein interactions across all the proteins (vignette 1, section O and vignette 2, section P).** Same as Figure 2 in the main manuscript, except the selection of temperatures for MSstatsTMT statistical model was centered around t ϵ {4,5,6}.

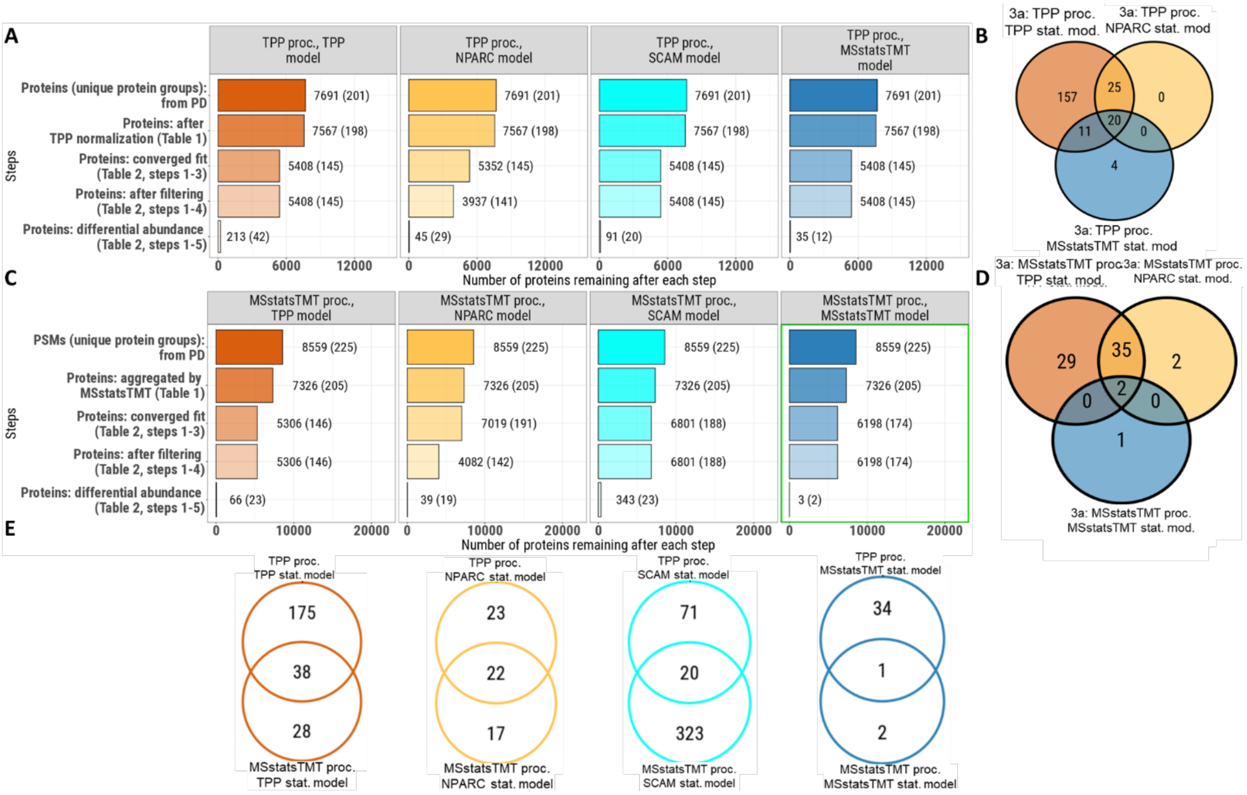

Figure S-27. **Dataset 3a- Xu *et al.* with contrasts set at mid temperatures (vignette 5 and vignette 6, section S).** Same as Figure 3 in the main manuscript, except the selection of temperatures for MSstatsTMT statistical model was centered around t ϵ {4,5,6}.

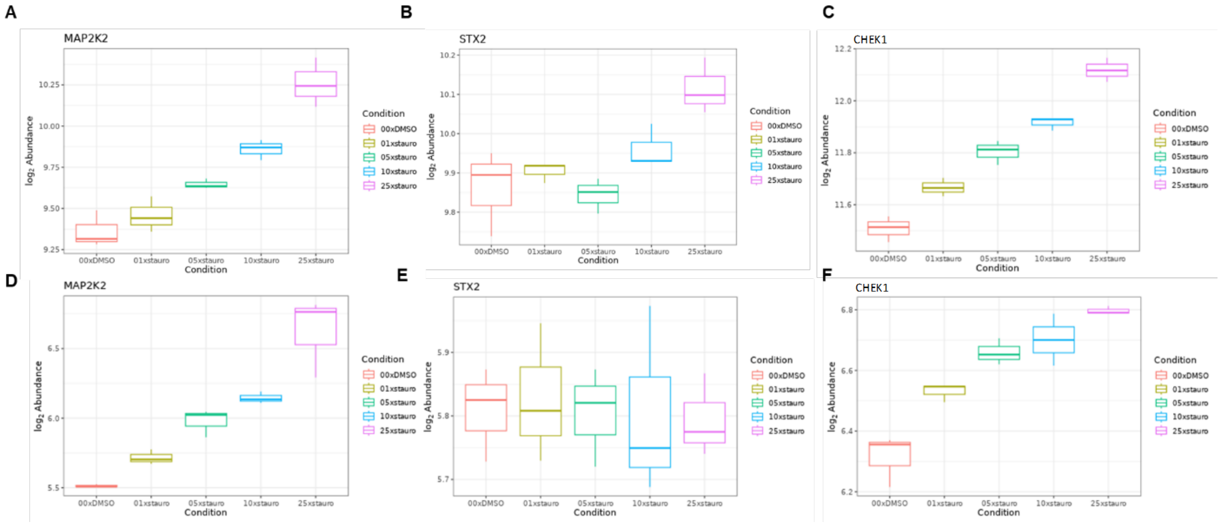

Figure S-28. **Dataset 3b-Xu *et al.* interacting proteins MAP2K2 and CHEK1 and non-interacting protein STX2 (vignettes 7 and 8).** A-C: PD-level protein interactors D-F) MSstatsTMT summarized protein interactors. Processing can influence dose-dependent trends.

1. Le Sueur, C.; Hammarén, H. M.; Sridharan, S.; Savitski, M. M., Thermal proteome profiling: Insights into protein modifications, associations, and functions. *Current opinion in chemical biology* **2022,** *71*, 102225.
2. Sueur, C. L.; Rattray, M.; Savitski, M., Hierarchical Gaussian process models explore the dark meltome of thermal proteome profiling experiments. *bioRxiv* **2023**, 2023.10.26.564129.
3. Xu, Y.; West, G. M.; Abdelmessih, M.; Troutman, M. D.; Everley, R. A., A Comparison of Two Stability Proteomics Methods for Drug Target Identification in OnePot 2D Format. *ACS Chemical Biology* **2021,** *16* (8), 1445-1455.
4. Phaneuf, C. G.; Aizikov, K.; Grinfeld, D.; Kreutzmann, A.; Mourad, D.; Lange, O.; Dai, D.; Zhang, B.; Belenky, A.; Makarov, A. A.; Ivanov, A. R., Experimental strategies to improve drug-target identification in mass spectrometry-based thermal stability assays. *Communications Chemistry* **2023,** *6* (1), 64.
5. Savitski, M. M.; Reinhard, F. B. M.; Franken, H.; Werner, T.; Savitski, M. F.; Eberhard, D.; Molina, D. M.; Jafari, R.; Dovega, R. B.; Klaeger, S.; Kuster, B.; Nordlund, P.; Bantscheff, M.; Drewes, G., Tracking cancer drugs in living cells by thermal profiling of the proteome. *Science (New York, N.Y.)* **2014,** *346* (6205), 1255784.
6. Childs, D.; Kurzawa, N.; Franken, H.; Doce, C.; Savitski, M.; Huber, W., TPP: Analyze thermal proteome profiling (TPP) experiments. *R package version* **2023,** *3.28.0* (0).
